# Seasonal dynamics and starvation impact on the gut microbiome of urochordate ascidian *Halocynthia roretzi*

**DOI:** 10.1186/s42523-020-00048-2

**Published:** 2020-08-18

**Authors:** Jiankai Wei, Hongwei Gao, Yang Yang, Haiming Liu, Haiyan Yu, Zigui Chen, Bo Dong

**Affiliations:** 1 Key Laboratory of Marine Genetics and Breeding, College of Marine Life Sciences, Ocean University of China, Qingdao, 266003 China; 2 Laboratory for Marine Biology and Biotechnology, Qingdao National Laboratory for Marine Science and Technology, Qingdao, 266237 China; 3 Institute of Evolution and Marine Biodiversity, Ocean University of China, Qingdao, 266003 China; 4 Technology Center of Qingdao Customs, Qingdao, 266002 China; 5 Department of Microbiology, Faculty of Medicine, The Chinese University of Hong Kong, Hong Kong, China

**Keywords:** Ascidian, Gut microbiome, Metabolites, Starvation stress, Environmental adaptation

## Abstract

**Background:**

Gut microbiota plays important roles in host animal metabolism, homeostasis and environmental adaptation. However, the interplay between the gut microbiome and urochordate ascidian, the most closet relative of vertebrate, remains less explored. In this study, we characterized the gut microbial communities of urochordate ascidian (*Halocynthia roretzi*) across the changes of season and starvation stress using a comprehensive set of omic approaches including 16S rRNA gene amplicon sequencing, shotgun metagenomics, metabolomic profiling, and transcriptome sequencing.

**Results:**

The 16S rRNA gene amplicon profiling revealed that ascidians harbor indigenous gut microbiota distinctly different to the marine microbial community and significant variations in composition and abundance of gut bacteria, with predominant bacterial orders representing each season. Depressed alpha-diversities of gut microbiota were observed across starvation stress when compared to the communities in aquafarm condition. Synechococcales involving photosynthesis and its related biosynthesis was reduced in abundance while the enrichments of Xanthomonadales and Legionellales may facilitate bile acid biosynthesis during starvation. Metabolomics analysis found that long chain fatty acids, linolenic acid, cyanoamino acid, and pigments derived from gut bacteria were upregulated, suggesting a beneficial contribution of the gut microbiome to the ascidian under starvation stress.

**Conclusions:**

Our findings revealed seasonal variation of ascidian gut microbiota. Defense and energy-associated metabolites derived from gut microbiome may provide an adaptive interplay between gut microbiome and ascidian host that maintains a beneficial metabolic system across season and starvation stress. The diversity-generating metabolisms from both microbiota and host might lead to the co-evolution and environmental adaptation.

**Graphical Abstract:**

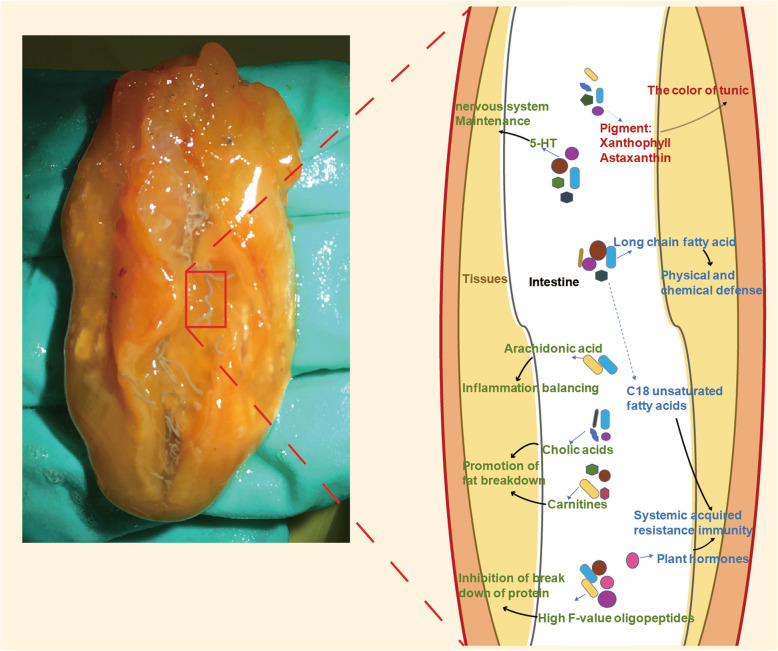

## Background

Ascidians, or sea squirts, are urochordate invertebrate that have been widely used as a model organism in developmental and evolutionary biology for decades [1]. Ascidians are also known as invasive organism forming biofouling in marine aquaculture that cases economic loss [2–4]. Natural selection in genomes of several marine animals, such as oyster and scallop, could play a role of molecular adaptation to marine environment [5, 6]. Recently, the genome of leathery sea squirt (*Styela clava)* has also been sequenced and analyzed for the understanding of their environmental adaptation [7]. However, the genetic basis driving ascidians to contribute and adapt marine ecosystem remains to be elusive. It has been reported that metabolic products in ascidians, such as alkaloids, cyclic peptides, and polyketides, could demonstrate high defensive bioactivity that may facilitate host ecological success in environmental invasion and adaptation [8–10]. These bioactive compounds could be synthesized by ascidians or their bacterial symbionts [11, 12]. For example, Ecteinascidin 743 (ET-743), a natural marine compound derived from the Caribbean sea squirt *Ecteinascidia turbinate* that has antineoplastic activity and is used to treat soft tissue sarcoma [13], was proved to be product of its commensal bacteria *Candidatus Endoecteinascidia frumentensis* [14]. Similarly, Didemnin B, isolated from bacterial strains *Tistrella mobilis* and *T. bauzanensis* in Caribbean ascidian *Trididemnum solidum* [15], exhibited high toxicity and has demonstrated impressive anticancer activity in preclinical models [16]. Increasing evidences indicate a defensive role of metabolic products from the symbiotic bacteria in ascidians [17], where the tunic of ascidians have been characterized as one of habitats colonized with high abundance of bacteria. For example, the 16S rRNA gene pyrosequencing revealed a high diversity of bacteria in the inner tunic of *Styela plicata* [18], *Ciona intestinalis* [19], and Great Barrier Reef ascidians [20]. The result of comparison among the different tunic-originated microbiomes in *C. intestinalis*, *C. savignyi*, *Botrylloides leachi* and *Botryllus schlosseri* showed that bacterial phylotype profiles were conserved within each species, and each species had a distinct set of bacterial OTUs (operational taxonomic units) [21]. The seasonal and spatial dynamics of the microbial communities in the inner-tunic of two invasive ascidians, *S. plicata* and *Herdmania momus* were also examined [22].

The gut harbors an enormous variety of microbiota that form a community playing important roles in host metabolism and immune system [23]. In mammals, the epithelial cells in the gut make up the mucosal interface between the host and microorganisms, by which microbial metabolic products gain access to and interact with host cells [24]. Those gut originated-microbial metabolic products play diversely unexpected roles in maintenance and regulation of host animal physiology and strengthening environmental adaptation [25]. In ascidians, the gut space is compartmentalized into a luminal part by envelope membranes. The membranes confined microbes to the luminal space and maintained the ciliated epithelium free of microbes [26]. The geographically disparate *C. intestinalis* was found to harbor a core microbiota in the gut [27]. Even the unique viral communities were identified in the gut of *C. intestinalis* [28]. Recent work revealed that ascidian microbiomes and metabolomes contain species-specific and location-specific components [29]. However, compared with other experimental models and ecologically important marine species, our knowledge on ascidian gut microbiome and the interplaying between bacteria and host remain largely unknown.

In this study, we investigated the community diversity and the dynamic changes of gut microbiota in sea squirt (*Halocynthia roretzi*) using multiple omic approaches including 16S rRNA gene amplicon sequencing, shotgun metagenomics, metabolomic profiling, and transcriptome sequencing. Our results showed that ascidian gut microbiota presented seasonal variation. Defense and energy-associated metabolites derived from the gut microbiome were upregulated when ascidians suffered starvation stress. The findings indicate a beneficial metabolic system and environmental adaptation the gut microbiota may have during the long history of bacteria and host co-evolution.

## Results

### Sample collection

**Fig. 1:**
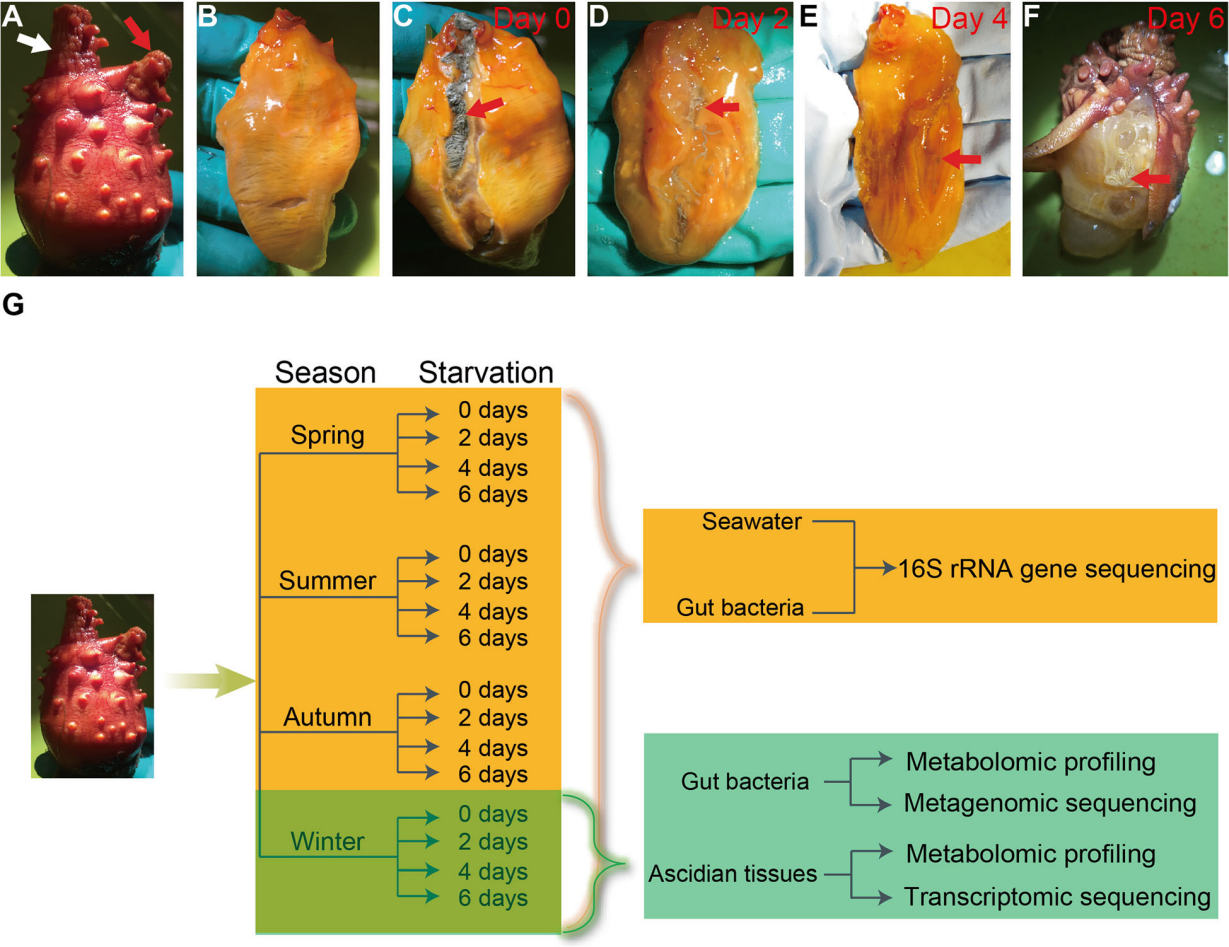
Sampling and experimental outline. **a** The adult *H. roretzi* used in this study. White and red arrows indicate the oral and atrial siphon, respectively. **b** The ascidian adult without tunic. **c**-**f** The stools and animals with starvation for 0, 2, 4, and 6 days, respectively. The red arrows indicate the stool inside the gut. Before starvation (Day 0), the stools are black, strip-shaped and curved (**c**). After starvation for 2 days (Day 2), the stool become thinner and fewer (**d**). After starvation for 4 days (Day 4), the stool becomes much thinner (**e**). After starvation for 6 days (Day 6), the stool becomes white and sticky (**f**). **g** The outlines of the sampling. Animals are starved for 0, 2, 4, and 6 days in January, April, July and October, respectively. The stools inside the gut and the seawater in the sampling locations are used for 16S rRNA gene sequencing. The stool samples in January are also utilized for metagenomic sequencing and metabolomics analysis, respectively. The tissues of the corresponding animals, which are sampled in January are used for RNA-Seq and metabolomics analysis

The living adults of ascidian (*H. roretzi*) were collected in four distinct months (January, April, July, and October 2018) that briefly represent four main seasons. No apparent morphological changes among animals from different seasons were observed (Fig. 1a and b). Usually the peritrophic membranes of ascidians formed long stringy shape twist filled with dark fecal materials (red arrow, Fig. 1c). However, the peritrophic membranes of the gut became lighter and slimmer, and were covered with sticky secretions without food supply for 2 days and longer (Fig. 1d-f).

Stool samples were collected to delineate the changes of gut microbiota by season, before and after starvation, using 16S rRNA gene amplicon sequencing (Fig. 1g). In order to further understand host-microbe interaction, the gut microbiome in Winter season (January 2018) were isolated for metabolite profiling. Meanwhile, stool samples and ascidian peritrophic tissue samples before (day 0) and after starvation (day 2, 4, and 6) in Winter season were conducted with shotgun metagenomic and transcriptomic sequencing for bacterial and host gene metabolic functional analysis, respectively (Fig. 1g).

### Ascidian gut microbiota compared with that of marine environment

**Fig. 2:**
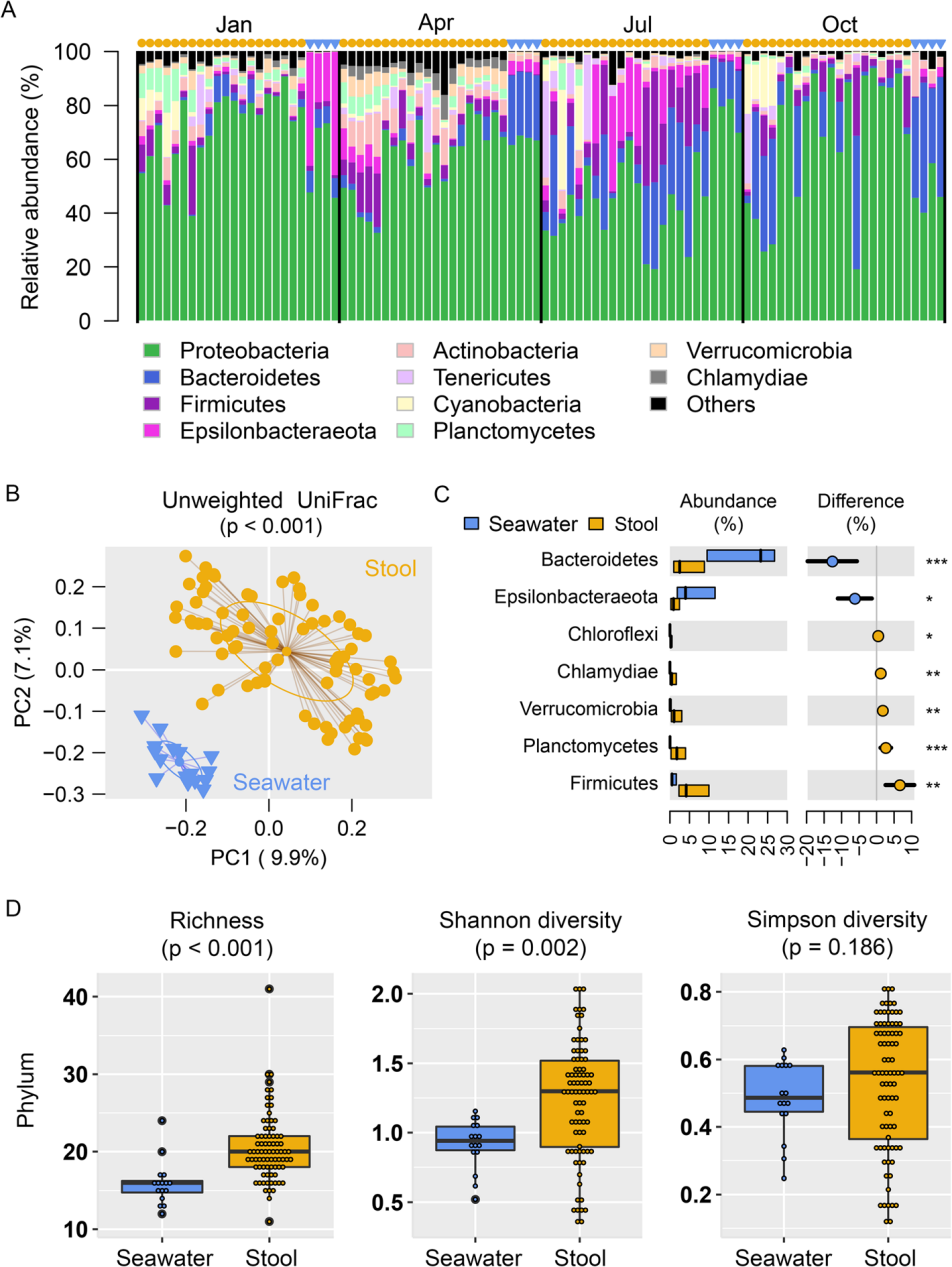
The composition and diversity of stool and seawater microbiota based on 16S rRNA gene sequencing. **a** The relative abundance of bacterial composition of the stool and seawater samples at the phylum level. The yellow dots above the bars indicated the results of stool samples while the blue triangle above the bars indicated the results of water samples. The top 10 phyla are labeled in different colors. Most of the samples are dominated by proteobacteria (labeled in green color). The stool displays different composition compared with the seawater samples in each season. **b** The clustering analysis of all the samples based on the unweighted UniFrac method. The stool samples are labeled in yellow dot, while the seawater samples are labeled in blue triangle. **c** The significantly different abundance between stool and seawater samples at the phylum level. The abundance is shown in columns while the difference is shown in dot. **d** The box plot of the richness, Shannon diversity, and Simpson diversity of stool and seawater samples. The stool samples show significantly high levels of richness and Shannon diversity

We first used 16S rRNA gene hypervariable V4 region amplicon sequencing to compare the difference of microbial communities between the gut and the marine environments. Four seawater samples in each season (*n* = 16) and five stool samples at each day timepoint of starvation (*n* = 80) were surveyed, with a total of 4,813,906 high-quality sequences generated from 96 samples (mean ± s.d. of 50,144 ± 9682). A rarefaction analysis of 20,000 reads per sample clustered short reads into 20,992 amplicon sequence variants (ASVs) that represented 54 bacterial phyla. Among them, 16 phyla were detectable at ≥1% relative abundance in at least one sample (Table S1). Proteobacteria (mean relative abundance of 61.1%) was the most predominant bacterial phylum in the surveyed samples, followed by Bacteroidetes (11.2%) and Firmicutes (6.5%) (Fig. 2a).

As expected, we observed differential bacterial communities between samples from seawater and ascidian peritrophic membranes, as discriminated by a principal coordinate analysis (PCoA) using either UniFrac distances or Bray-Curtis dissimilarities (Fig. 2b and Fig. S1). A permutational multivariate analysis of variance (PERMANOVA) using the *adonis2* function in R’s package ‘vegan’ based on unweighted UniFrac distances (mean distance between seawater and stool = 0.0531; *p* < 0.001) found a more distinct discrimination in microbial community composition when compared to the weighted UniFrac distances (0.0497; *p* = 0.003) (Figure S1), indicating that the clustering between ascidian gut and marine seawater samples was driven more by the presence/absence of bacterial ASVs (unweighted) rather than the proportion of microbial community members (weighted). For example, a significant increase of the relative abundance of Bacteroidetes and Epsilonbacteraeota were observed in seawater (Fig. 2c, Table S1) whereas Firmicutes was more common in ascidian stool samples (Fig. 2c). When ASVs were summarized at the order levels, Flavobacteriales, Oceanospirillales, Alteromonadales, and Campylobacterales were largely observed in seawater (mean relative abundance > 5%, MWU *p* < 0.002), while ascidian stool samples were mainly dominated by Xanthomonadales, Rhizobiales, Legionellales, and Bacteroidales (Table S2), indicating that the bacterial communities may form the strong niche adaptation. In line with differential compositions and abundances, the microbial community of ascidian stool samples showed higher alpha diversities when compared to the seawater (Fig. 2d and Figure S2).

### Ascidian gut microbiota changed by season and starvation stress

**Fig. 3:**
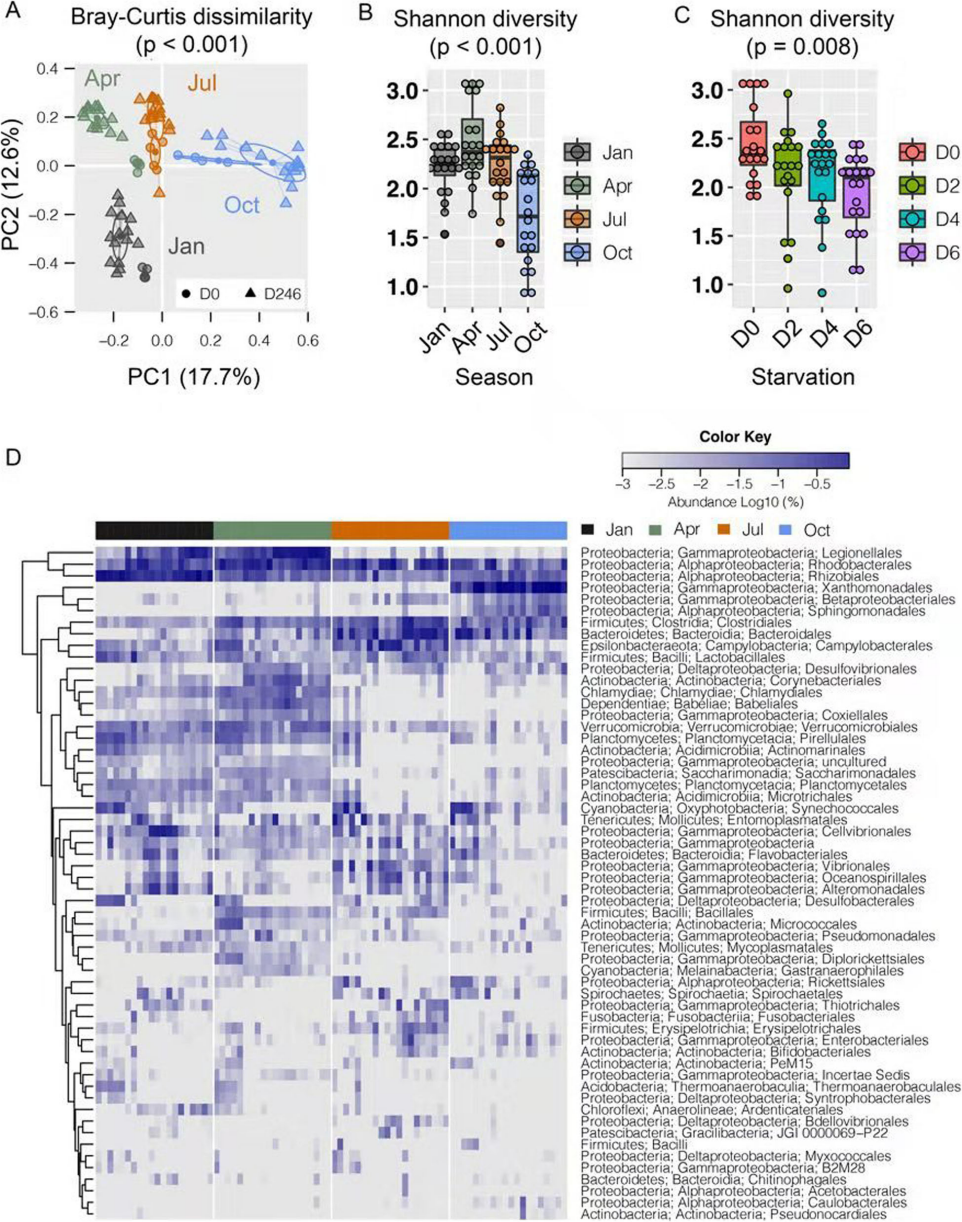
The variation of bacterial abundance and composition of stool samples along with seasons. **a** The clustering analysis of all stool samples based on Bray-Curtis method. The samples in different seasons are labeled in different colors. **b** The Shannon diversity of stool samples in different seasons. The samples in April has the highest value while the samples in October has the least value. **c** The Shannon diversity of stool samples under different starvation days. The samples in Day 0 has the highest value while the samples in Day 6 has the least value. **d** The heatmap of the bacterial abundance of stool samples at the order level. The order names of each row are shown on the right of the heatmap

In order to elucidate the changes of ascidian gut microbiota by season and starvation stress, we refined the ASV table by excluding the seawater samples. Overall, ascidian gut microbiota was mainly dominated by Proteobacteria (mean relative abundance of 46%, represented by Rhodobacterales, Xanthomonadales, Rhizobiales, and Legionellales), followed by Bacteroidetes (8%, represented by Bacteroidales) and Firmicutes (5%, represented by Clostridiales) (Table S3). A PERMANOVA test using Bray-Curtis dissimilarities based on the ASV table indicated that approximately 54% of variation in microbial community composition could be attributed to season (Df = 3, *R*^*2*^ = 0.359, pseudo F = 18.843, *p* < 0.001), starvation (Df = 1, *R*^*2*^ = 0.080, pseudo F = 12.609, *p* < 0.001) and the combination of season and starvation (Df = 3, *R*^*2*^ = 0.103, pseudo F = 5.384, *p* < 0.001), which was supported by the PCoA analysis that the majority of microbial variability was associated with differences between seasons (Fig. 3a). Similarly, we found significant changes of the alpha diversities of gut microbial communities across season (Fig. 3b) and starvation (Fig. 3c).

**Fig. 4:**
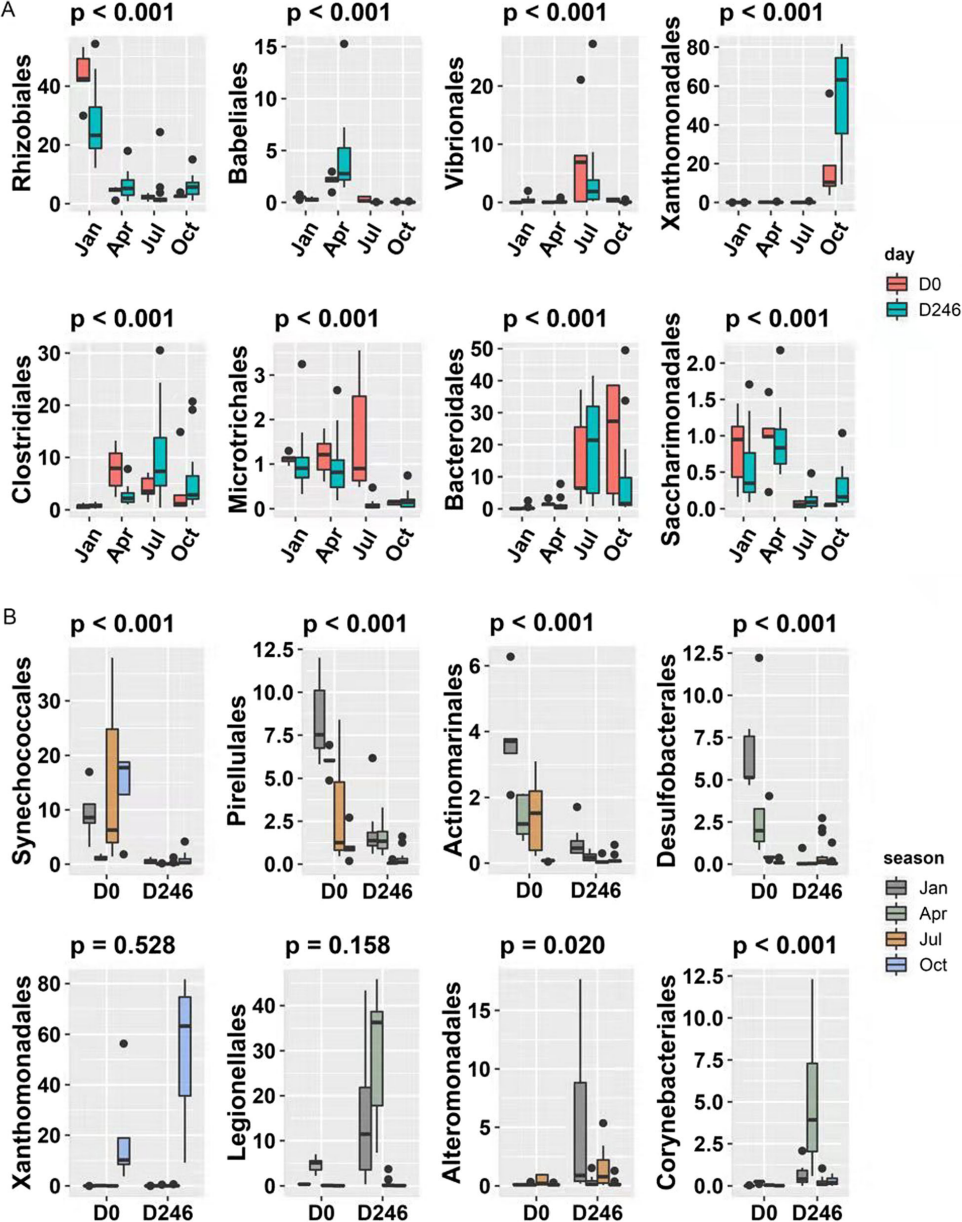
The dynamics of bacteria abundance along with different seasons and starvation at the order level. **a** The samples are divided into four seasons according to the sampling date. Rhizobiales has high abundance in January; Babeliales has high abundance in April; Vibrionales has high abundance in July; Xanthomonadales has high abundance in October. Clostridiales has high abundance in April, July, and October, while Microtrichales has high abundance in January, April, and July. Bacterioidales has high abundance in both July and October, and Saccharimonadales has high abundance in both January and April. **b** The samples are divided into two stages (Day 0 and Day 246) according to the starvation treatment. Synechococcales, Pirellulales, Actinomarinales, and Desulfobacterales have high abundance in Day 0 samples (without starvation). Xanthomonadales, Legionellales, Alteromonadales, and Corynebacteriales have high abundance in Day 246 samples (along with starvation for two, four, and 6 days, respectively)

The relative abundance analysis of bacterial orders revealed that ascidian gut microbiota presented seasonal variation (Fig. 3d and Figure S3, Table S3). For example, Rhizobiales was highly abundant in stool samples collected in January but rarely observed in other seasons (Fig. 4a). Babeliales, Vibrionales, and Xanthomonadales seemed to uniquely form dominant population in April, July, and October, respectively (Fig. 4a). In contrast, the colonization of some bacterial orders might be season-specific. For example, stool samples collected in January and October contained extremely low proportion of Clostridiales and Microtrichales, respectively (Fig. 4a). Bacteroidales and Saccharimonadales were rarely found in Jan/Apr and Jul/Oct, respectively. Interestingly, Xanthomonadales was commonly found in both ascidian stool samples (46.2% vs 0.1%, *p* < 0.001) and seawater (6.7% vs 0.1%, *p* < 0.001) collected in October but not in other seasons, implying that gut bacterial transmission from marine environment is possible (Figure S4, Table S4).

Consistent with the decreased alpha diversity of gut microbiota during starvation (Fig. 3c), a number of microbes largely changed in the relative abundances (Figure S5, Table S3). We found 13 bacterial orders prevalently decreased across starvation while another 11 becoming more resistant, with statistical significance in at least one season. As shown in Fig. 4b, for example, Synechococcales and Pirellulales, two predominant gut bacterial orders in aquafarm condition in most of seasons, were dramatically depressed when food and nutrition elements were lacking (mean relative abundance of 9.9% vs 0.4%, q < 0.001; 4.7% vs 0.9%, q < 0.001). In contrast, some rare bacteria in certain seasons, such as Xanthomonadales, Legionellales, Alteromonadales, and Corynebacteriales, became booming in starvation condition. The relative abundance analysis of bacterial genus also revealed similar seasonal variation (Table S5).

### Functional profile of ascidian gut microbiota based on 16S rRNA gene amplicon sequencing

Differential gut microbial communities observed between habitats, seasons, and starvation conditions indicates that these factors may enrich for functionally different microbial communities. Hence, we used PICRUSt2 (Phylogenetic Investigation of Communities by Reconstruction of Unobserved States) to predict functional pathways based on the composition of the microbial communities and produced Kyoto Encyclopaedia of Genes and Genomes (KEGG) Orthology (KO) abundance profiles. Results of the summarized KO pathways were supported by spare partial least squares discriminant analysis (sPLSDA) using the first three ordination components that show clustering of samples mainly by seasons and habitats (Figure S6).

**Fig. 5:**
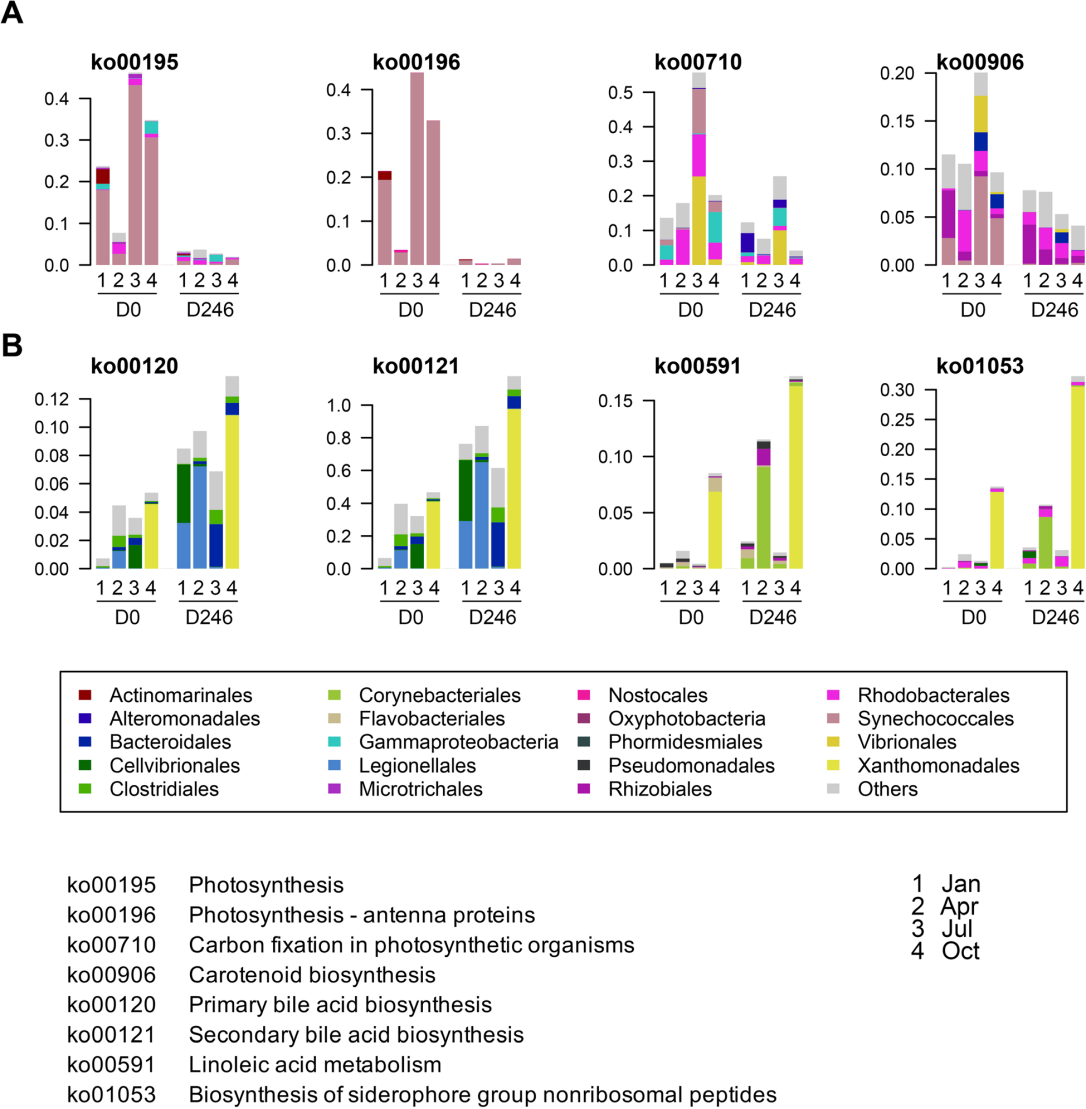
The significantly discriminated KO pathways before and after starvation for all seasons. The x axis indicated different samples and the y axis indicated the relative abundance of the bacteria. The different colors indicated different bacteria orders and the order names were listed in the rectangular box. The ko pathway annotations were listed at the bottom. **a** The decreased metabolism pathways along with starvation for all seasons. **b** The increased metabolism pathways along with starvation for all seasons

Next, we attempted to identify the metabolic functions that discriminated the ascidian gut microbial communities before and after starvation (Fig. 5a). As shown in Table S6, we found 26 up- and 22 down-regulated pathways across starvation, with statistical significance in one season and more. Among them, the functions involving photosynthesis (ko00195, ko00196) and its related biosynthesis (ko00710, ko00906) were dramatically depressed (baseMean > 1000, |log2FoldChange| > 1, q < 0.001) (Fig. 5a), probably a result of the reduced colonization of Synechococcales in the starvation condition (Fig. 4b). In contrast, enrichments of Xanthomonadales and Legionellales in relative abundances after starvation might facilitate bile acid biosynthesis (ko00120, ko00121) (Figs. 4b and 5b). Xanthomonadales and Corynebacteriales might also contribute linoleic acid metabolism (ko00591) and biosynthesis of siderophore group nonribosomal peptides (ko01053). The moderately increased metabolism pathway involving bacterial secretion system (ko03070, baseMean = 25,600, log2FoldChange = 0.36, q = 0.001) might explain in part the observation of sticky secretions covering the surface of ascidian peritrophic membranes during the starvation (Table S6). It is worth noting, however, that the limited resolution of partial 16S rRNA gene in discriminating bacterial phylotypes, as well as a possible lack of marine animal PICRUST2 reference microbial genomes may have limited resolution of functional prediction, given the relatively high scores of the weighted Nearest Sequenced Taxon Index (0.17 ± 0.10).

### Metabolic changes of gut microbiome and host across starvation

**Fig. 6:**
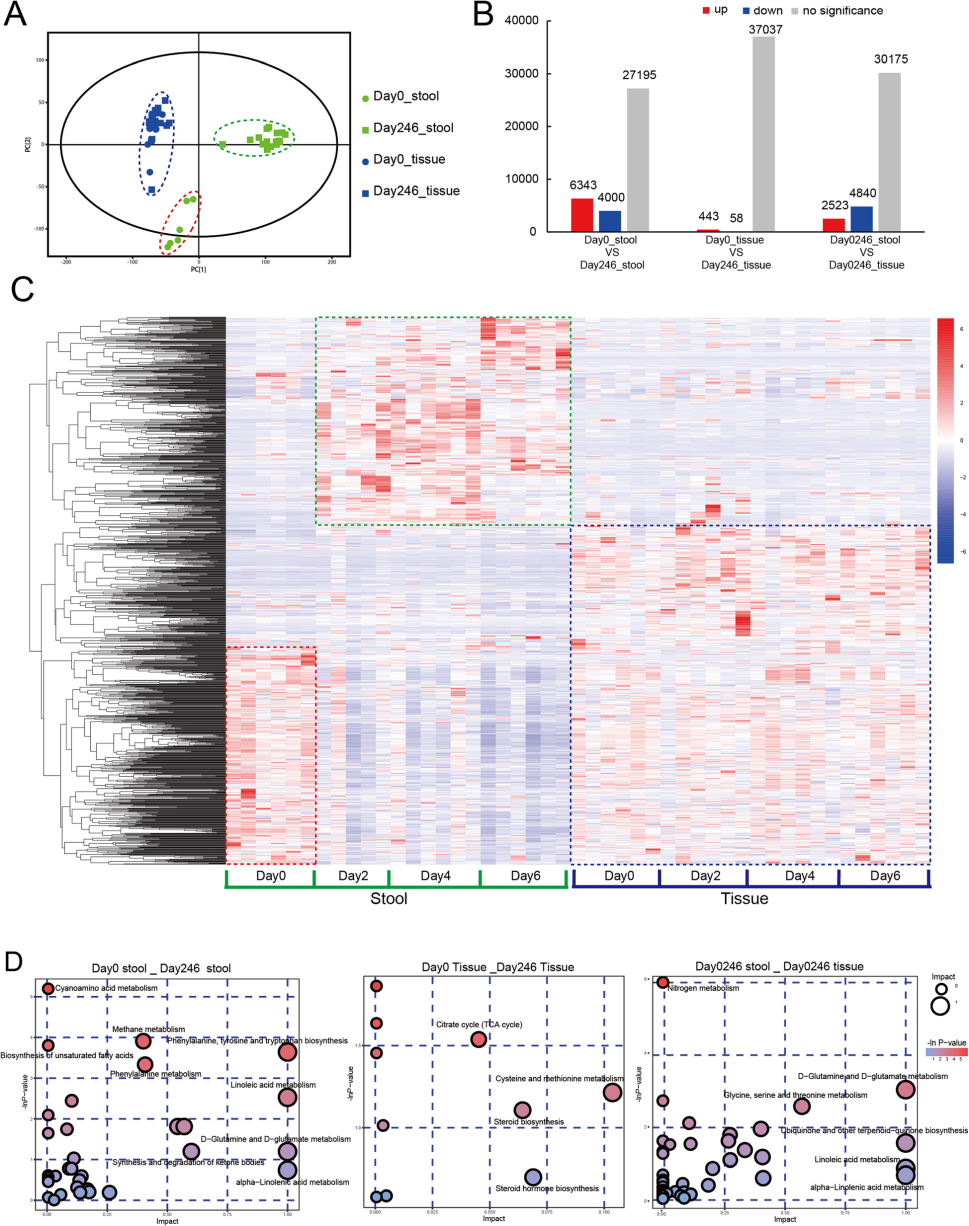
Metabolites composition and the differential analysis in both stool and ascidian tissue samples. **a** The PCA analysis of all the samples according to the abundance of metabolites. The green dots indicate stool samples before starvation (Day 0 stool); the green rectangles indicate stool samples with starvation for 2, 4, and 6 days (Day 246 stool); the blue dots indicate the tissue samples from *H. roretzi* before starvation (Day 0 tissue); the blue rectangles indicate tissue samples with starvation for 2, 4, and 6 days (Day 246 tissue). **b** The number of the differentially expressed metabolites between different groups. The red columns indicate the number of upregulated metabolites and the blue columns indicate the number of downregulated metabolites. **c** The abundance heatmap of the metabolites identified from both stool and tissue samples. The red color indicates the relatively high expression while the blue color indicates the relatively low expression. **d** The bubble plots of significantly different metabolites enriched pathways between different groups. The x-axis indicates the impact and the y-axis indicates the -ln *P*-value

In order to further understand the host-microbe interaction, the ascidian stool samples and peritrophic tissues collected in January were conducted for metabolic profiling using high-performance liquid chromatography. Among 37,538 identified metabolites, 1157 of them could be annotated as known ones using mass spectrometry data (MS2 spectrum) and metabolic reaction network (MRN)-based recursive algorithm (MetDNA) (Table S7). The PCoA analysis based on the abundance of all the identified metabolites clearly discriminated stool samples from the ascidian tissues (Fig. 6a), implying differential metabolic profiles between microbiota and host. We also observed distinct separation of stool samples before (Day 0) and after starvation (Day 246). However, the metabolic profiles of the ascidian tissues did not significantly change before and after starvation, suggesting that starvation mainly has significant impact on the gut microbiome rather than host (Fig. 6b, Table S8). When the abundances of metabolites were visualized in a heatmap, we observed a pattern of metabolites highly expressed in stool samples across starvation when compared with those in aquafarm condition (see green rectangle in Fig. 6c), such as the pathways involving linolenic acid metabolism, methane metabolism, and cyanoamino acid metabolism (Fig. 6d). In contrast, a number of abundant metabolites in aquafarm condition were dramatically depressed (see red rectangle in Fig. 6c), such as phenylalanine metabolism, phenylalanine tyrosine, tryptophan biosynthesis, and D-glutamine and D-glutamate metabolism (Fig. 6d). Some metabolites might be host- or bacteria-specific. For example, linoleic acid, a product from plants and green algae, could also been synthesized by bacteria [30–32]. Interestingly, there was limited impact of starvation in regulating metabolites of host tissue samples implying that the dysbiosis of gut microbiome may be mainly responsible for the changes of metabolites across starvation.

### Contribution of gut microbiome in metabolite changes

To determine the influence of gut microbiome in changing metabolic pathways before and after starvation, we first performed the correlation analysis of metabolites, and found that the abundance between bacteria and metabolites were highly correlated. (Figure S7), suggesting bacterial origin of metabolites. For example, phosphatidylcholine lyso and arachidonate in arachidonic acid metabolism pathway were highly correlative with Rhodobacteriales, Flavobacteriales, vibrionales, and Spirochaetales etc. (Figure S7).

To further reveal the origin and the difference of metabolites between ascidian gut microbiome and peritrophic tissue, transcriptome sequencing for tissue samples (*n* = 4) (Table S9) and metagenomic sequencing for stool samples (*n* = 4) (Table S10) in winter, before and after starvation, were performed, respectively. A total of 176.6 and 242.3 million short reads were obtained from transcriptome and metagenome sequencing, respectively. One hundred and eighteen KEGG pathways were shared by both stool and tissue samples across metabolome and metagenomic analyses (Figure S8A). Interestingly, 83 pathways (9 + 66 + 8) observed in either stool or tissue samples with metabolome analysis were detectable in the metagenomic annotation of gut microbiome but not peritrophic tissue, indicating that the gut microbiome largely contribute the synthesis and decomposition of metabolites, such as alox15 and beta-carotene 3-hydroxylase (Figure S8B and S8C).

**Fig. 7:**
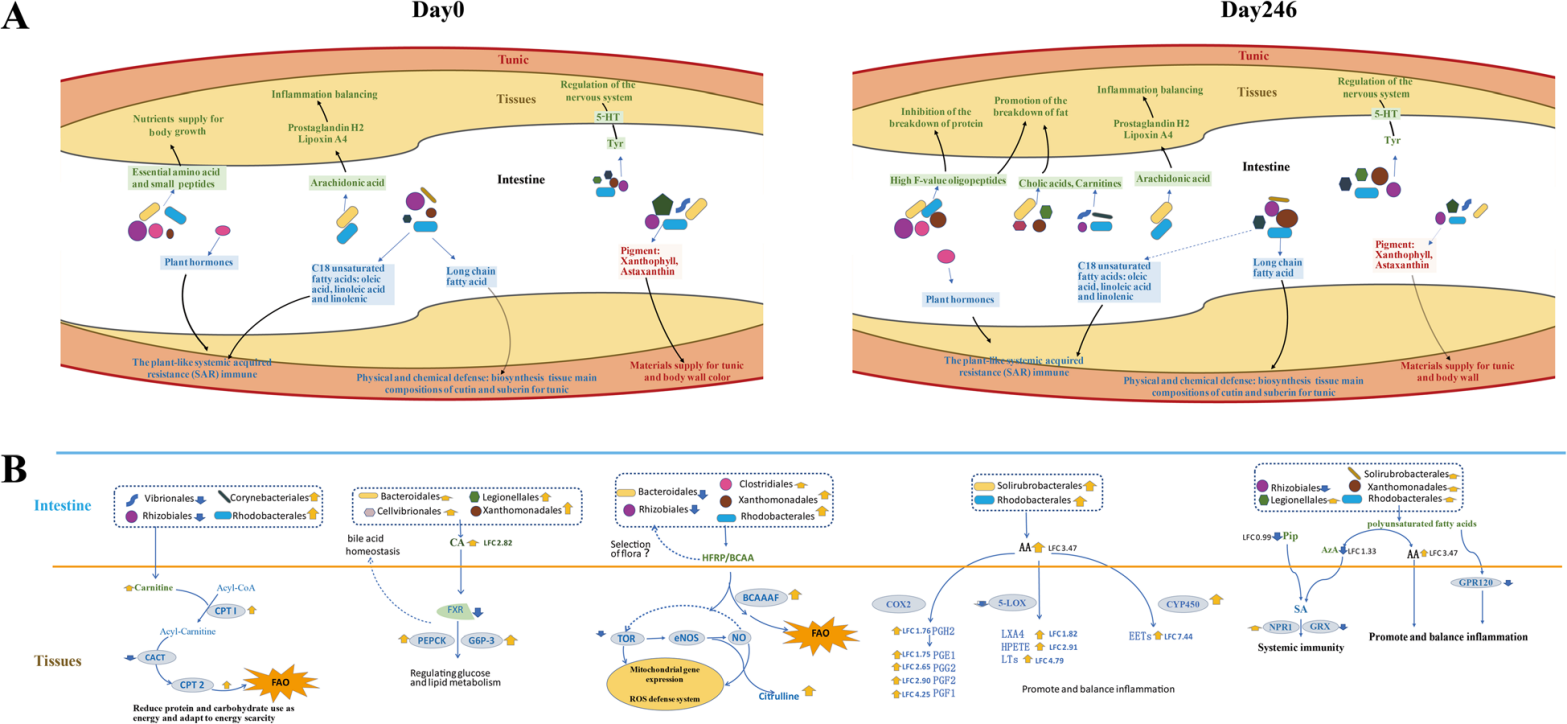
The mutually beneficial model between gut microbiota and host in ascidian *H. roretzi*. **a** The enriched metabolites in stool samples and their potential connection with host tissues without (Day 0) or with (Day 246) starvation stress. The metabolites include the pigment compounds astaxanthin and Xanthophyll, plant-like polyunsaturated fatty acids and esters, hormone signal substance, plant hormones (such as salicylic acid and stearidonic acid), C18 unsaturated fatty acids, phenylalanine, benzoate, salicylic acid, and stearidonic acid. These gut bacteria-originated metabolites likely play crucial roles on energy supports, inflammation balancing, and body defense through glucose and lipid metabolism pathways. **b** The putative mechanisms of the interactions between gut microbiota and host ascidians. The dashed box indicates the putative source bacteria of the metabolites. The yellow arrows indicate the upregulation of bacteria, metabolites or gene expression under starvation stress. The blue arrows indicate the downregulation of bacteria, metabolites, or gene expression under starvation stress

Potential metabolic pathways involving the interactions between ascidian gut microbiome and host were proposed. As observed, the pigment compounds (such as astaxanthin and Xanthophyll), plant-like polyunsaturated fatty acids and esters, hormone signal substance, plant hormones (such as salicylic acid and stearidonic acid), C18 unsaturated fatty acids (such as oleic acid, linoleic acid, and linolenic), phenylalanine, benzoate, salicylic acid, and stearidonic acid were significantly enriched in the gut (Fig. 7a). These bacterial origin metabolites were likely absorbed and played crucial roles on host energy supports, inflammation balancing, and body defense through glucose and lipid metabolism pathways. For example, plant hormones and C18 unsaturated fatty acids are common signaling substances constituting the systemic acquired resistance (SAR) immune system in ascidian gut (Fig. 7a). In contrast, unsaturated fatty acid-related metabolism including arachidonic acid and linoleic acid was significantly enhanced at Day 246, suggesting that the gut microbiome may serve as nutritional supplements involved in body functions when ascidian is under the starvation stress (Fig. 7a). A number of gut bacterial orders were deduced as metabolic contributors (Fig. 7b). For example, Rhodobacterales and Xanthomondadales may produce carnitine, cholic acid (CA), and branched-chain-amino-acids (BCAA) that regulate glucose and lipid metabolism for energy maintenance; Solirubrobacterales and Rhodobacterales could be the source bacteria related to inflammation balancing and systemic immunity (Fig. 7b).

## Discussion

In this work, we applied multiple omics approaches to characterize the dynamic change of gut microbiota of ascidian *H. roretzi* throughout the season and under starvation conditions. Indigenous gut microbiota was observed in ascidians, which is significantly different from marine communities. Many gut bacteria may firmly colonize in the digestive tract in the form of symbiotic diazotroph, such as Bacteroidales, that plays important roles in nitrogen metabolism [33, 34]. Synechococcales represents a group of cyanobacteria and was enriched in the gut of ascidians. It has been reported that cyanobacteria and ascidian host have a symbiosis relationships: cyanobacteria symbionts provide nutriments and participate in defense for the ascidian host by means of carbon fixation, nitrogen recycling, and metabolites production [35]; meanwhile, ascidian can provide nitrogen nutrients for the growth of cyanobacteria symbionts and protect them from ultraviolet radiation [36]. We observed seasonal changes in the gut microbiota of ascidians that was not related to diet. Starvation is another environmental factor that seriously affects the gut microbial community. The dynamic changes of ascidian gut microbiota before and after starvation subsequently changed the metabolism and related pathways, thereby providing an adaptive interaction and a beneficial metabolic system between the gut microbiome and the ascidian host.

The gut microbiota may promote the stable metabolic system in ascidians. During starvation, we observed an increase in the abundance of gut metabolites, which are part of glucose, lipid, and peptide metabolic pathways mainly contributing to energy supply, inflammation balance and immune defense. The key synthetic or degraded enzymes involved in these pathways are usually rare in the ascidian digestive tract, indicating that the gut bacteria are the main source of metabolites. For example, our data showed that several pigment molecules in ascidian gut metabolites were significantly higher than those in the host tissues, such as astaxanthin and Xanthophyll. The transcriptome data showed that gut bacteria had the main enzymes of these pigment synthesis pathways, whereas, there was no enzyme of these pigment pathways in the ascidian. These results suggested that pigments are mainly from gut bacteria. They might contribute to the formation of tunic color through the transportation cross the tissues. Various long-chain fatty acids in gut bacteria were significantly higher than those in the host. These long chain fatty acids included docosahexaenoic acid, eicosadienoic acid, heneicosanoic acid, behenic acid, pinolenic acid. Significantly higher phospholipids in the gut included C16 Sphinganine, Sphingosine 1-P, Phytosphingosine, and Trimethyl-sphingosine. These long-chain fatty acids are raw material of cutin and suberin [37–40]. Phospholipids are raw material of cutinase [41, 42]. The transcriptome data of the ascidian tissues showed that the ascidian had no enzyme that are reqired for the long-chain fatty acid synthesis pathways and phospholipid pathways, while the microorganisms in ascidian gut had. The metabolome data showed that the gut microbes also synthesized higher content of primary bile acids and secondary cholic acids, including cholic acid, lovastatin acid, bisnorcholic acid, podecdysone B, cholesterol, pregnenolone, epiandrosterone, endrosterone, prostaglandins, which played synergistic roles in transporting long-chain fatty acids in ascidian tissue and regulating the metabolism of long-chain fatty acids [43, 44]. Therefore, long-chain fatty acids and phospholipids were speculated to be synthesized by gut microbes and then be transported through the body tissues to the tunic to synthesize cutin and cutinase. The fatty acid was a synthetic raw material of cutin and suberin [45, 46]. Cutin and suberin could inhibit the spore germination of fungi [47], and the unsaturated fatty acids could reduce the attachment of the surface of organisms [48]. Cutin and suberin could also protect the ascidian from being swallowed by other marine organisms [49, 50]. In addition, the elevated secondary bile acids may contribute to the control of harmful bacteria [51].

In addition, it seems that ascidian gut microorganisms could directly synthesize some plant-type and insect-type hormones, such as salicylic acid, stearidonic acid, juvenile hormone I, podecdysone B, Iloprost, Nicotinamide. Those plant hormones could induce the wound defense against diseases [52, 53], and those insect hormones were involved in improving chemical defense and immunity [54–57]. Whether these phytohormone receptors were presented on the ascidian itself or on other commensal bacteria was still unknown.

Our data also showed that the gut of the ascidian contained high levels of the arachidonic acid, lipoxinA4, and 5-Hydroxytryptamine (5-HT, serotonin). These substances were secreted into the gut by intestinal cells under the stimulation of gut microbes [58–60]. The arachidonic acid has an immune-enhancing effect [61]. Enteroendocrine cells within the mucosal lining of the gut synthesize and secrete a number of hormones including 5-HT, which have regulatory roles in key metabolic processes such as insulin sensitivity, glucose tolerance, fat storage, and appetite [58, 62, 63]. Gut-derived 5-HT in shaping gut microbiota composition in relation to susceptibility to colitis, identifying 5-HT-microbiota axis as a potential new therapeutic target in intestinal inflammatory [64]. The anti-inflammatory activities exhibited by the arachidonate metabolite lipoxin A4 was useful in downregulating active inflammation at mucosal surfaces [60].

## Conclusions

In conclusion, our data suggest that the biosynthesis of primary metabolites from gut microbiota provide as substance sources for host metabolism. Meanwhile, the metabolites secreted from hosts can also provide as substrates for microbiota such as galactinol and creatine, or contribute to the homeostasis of gut microenvironment such as serotonin. The diversity-generating metabolisms from both host and microbiota might lead to the co-evolution and environmental adaptations.

## Materials and methods

### Animal ethics approval

This study has been approved by the Ocean University of China Institutional Animal Care and Use Committee (OUC-IACUC). All experiments and relevant methods were carried out in accordance with the approved guidelines and regulations of OUC-IACUC.

### Sample collection

The living adults of ascidians (*H. roretzi*) were collected from an aquafarm in Weihai City, Shandong province, China in January, April, July, and October 2018, respectively. These animals were attached along the rope in the outdoor sea water. The adult ascidians were used for sampling. Animals were dissected immediately (day 0) or starved in filtered seawater at 18 °C without food for 2, 4, and 6 days and then dissected. Stool samples from peritrophic membranes were freshly frozen in liquid nitrogen until further DNA extraction. For each day timepoint, at least five ascidian animals were randomly selected for dissection. In order to compare the difference of microbial communities between the collections from animal hosts and the aquatic environment, seawater from the same aquatic sites was sampled. In brief, for each season at the same day when ascidians were collected, one liter of seawater from four sites around 1 m distant to each other were filtered through 200 μm membrane; membranes were stored in liquid nitrogen until further DNA extraction. Meanwhile, the tissues of ascidians collected in January were freshly frozen in liquid nitrogen for further host gene transcriptomic and metabolomic analyses.

### Microbial 16S rRNA gene sequencing and bioinformatics

Microbiota from stool samples or membranes were extracted for total DNA using CTAB method. The 16S rRNA gene hypervariable V4 region was amplified using primers 515F (5′- GTGYCAGCMGCCGCGGTA − 3′) and 806R (5′- GGACTACNVGGGTWTCTAAT − 3′) indexed with a pair of dual barcodes on both primers, and then sequenced using Ion S5 System (Thermofisher, USA). Following demultiplexing, the QIIME2 (2019.1) package [65], including pipelines for quality control, dada2 denoising and sequence clustering, was applied to assign 16S rRNA gene short reads into amplicon sequence variants (ASVs) table. Singleton reads were removed. The SILVA v132 99% 16S rRNA gene reference database (https://www.arb-silva.de/download/archive/qiime) was used to classify and summary ASVs with bacterial taxonomy by proportion at different levels including genus, family, order, class and phylum ranks.

In order to retain all samples for diversity analysis, reads from each sample were rarefied to the depth of 20,000 to normalize the data for differences in sequence count. GUniFrac (unweight or weight) pairwise distances or Bray-Curtis dissimilarities between samples were calculated using scripts in R v3.4.0 package. Differences in community composition were assessed using permutational multivariate analysis of variance (PERMANOVA) in the Vegan R package. Principal coordinate analysis was performed to visualize associations between community composition and experimental factors. Comparisons of the relative abundances of ASVs between defined groups were performed using nonparametric Mann-Whitney Wilcoxon rank sum test (MWU), Kruskal-Wallis test (KW), or Tukey’s honest significant difference (Tukey HSD) post hoc test. A two-sided *P* value of < 0.05 was considered statistically significant.

### Functional prediction based on 16S rRNA gene community composition

Functional profiles of microbial communities were predicted using PICRUSt2 (https://github.com/picrust/picrust2/wiki) based on 16S rRNA gene sequences represented as Kyoto Encyclopedia of Genes and Genomes (KEGG) Orthology (KO) counts [66, 67]. These counts were summarized into KO hierarchies and then normalized in percentage for analysis. Metabolic pathways that discriminated between seasons and habitats were identified using sparse partial least squares discriminant analysis (sPLSDA) implemented in the mixOmics R package [68]. Differential abundances of KO pathways for the comparison between conditions were analyzed using the DESeq2 R package [69].

### Microbial and host tissue metabolomes analysis and statistical test

Each sample from gut stools or ascidian tissues in winter was added the extraction liquid containing an internal target, then homogenized in ball mill for 4 min at 45 Hz and ultrasound treated. After incubation for 1 h at − 20 °C to precipitate proteins and centrifuged at 12000 rpm for 15 min at 4 °C, the supernatant was transferred into a fresh 2 mL LC/MS glass vial for the UHPLC-QTOF-MS analysis. High Pressure Liquid Chromatography (LC) / mass spectrometry (MS) data were acquired using Q Exactive Orbitrap (Thermo Fisher Scientific, USA) coupled with Agilent 1290 HPLC system (Agilent, USA). Both positive ion mode (POS) and negative ion mode (NEG) were used for compound identification. MS raw data files were converted to the mzXML format using MSconventer, and processed by R package XCMS (version 3.2). MS2 database was applied in metabolites identification. The metabolic reaction network-based recursive algorithm (MetDNA) method was used to expand the metabolite annotations. Principal component analysis (PCA) were used to show the original data distribution. The OPLS-DA model was used with the first principal component of variable importance in projection (VIP) values (VIP > 1) combined with t-test (*P* < 0.05) and |log2FoldChange| > 1 to determine the significantly different metabolites among the pairwise comparison groups. Kyoto Encyclopedia of Genes and Genomes (KEGG, http://www.genome.jp/kegg/) was utilized to search for the metabolite pathways. MetaboAnalyst (https://www.metaboanalyst.ca/) was used for the pathway analysis.

### Microbial whole genome shotgun metagenomic sequencing and bioinformatics

The microbial DNA was conducted for shotgun metagenomic sequencing following standard library preparation using NEBNext Ultra DNA Library Prep Kit for Illumina (NEB, USA) following the manufacturer’s recommendations. The metagenome sequencing reads were acquired from Hiseq (Illumina, USA) platform. The raw reads were then preprocessed by removing reads with adaptors, reads with unknown nucleotides larger than 10 bps and reads with low quality (more than 40 bps with Q < 38). The pair end reads were assembled using SOAPdenovo software [70]. The gene prediction was conducted using MetaGeneMark software [71] and the taxonomy prediction was conducted by DIAMOND software [72].

### Host tissue transcriptome sequencing and bioinformatics

The preserved gut tissues were used for RNA extraction. The total RNA was extracted by RNAiso plus reagent (Takara, Japan), following the manufacturer’s instructions. RNA-seq were performed using the Illumina Hiseq 2500 platform. The raw reads were then preprocessed by removing reads with adaptors, reads with unknown nucleotides larger than 10% and reads with low quality (more than 50% of the bases with Q < 20). Sequencing reads were assembled by Trinity program [73]. The unigenes were then annotated by blast alignment [74] against nr, nt, swiss-prot, KEGG and COG databases. The gene expression levels were reflected by FPKM value of unigenes.

## Supplementary information

**Additional file 1.**

## Data Availability

The16S rRNA sequencing data were deposited in NCBI SRA database with the accession number of SRR10746888 to SRR10746983. The transcriptome data and metagenome data were also deposited in NCBI SRA database with the accession number of SRR10743032 to SRR10743035 and SRR10746995 to SRR10746999.

## Abbreviations

ET-743: Ecteinascidin 743
ASV: Amplicon sequence variant
PCoA: Principal coordinate analysis
PERMANOVA: Permutational multivariate analysis of variance
PICRUSt2: Phylogenetic Investigation of Communities by Reconstruction of Unobserved States
KEGG: Kyoto Encyclopaedia of Genes and Genomes
KO: KEGG Orthology
sPLSDA: Spare partial least squares discriminant analysis
MetDNA: Metabolic reaction network-based recursive algorithm
SAR: Systemic acquired resistance
CA: Cholic acid
BCAA: Branched-chain-amino-acid
5-HT: 5-Hydroxytryptamine
PCA: Principal component analysis
VIP: Variable importance in projection
