# Supplementary material for: Seasonal dynamics and starvation impact on the gut microbiome of urochordate ascidian Halocynthia roretzi
Source: Anim Microbiome. 2020 Aug 18;2:30. doi: 10.1186/s42523-020-00048-2 (PMC7807810; doi:10.1186/s42523-020-00048-2)
Supplement: Supplementary file 1 — Additional file 1. [file 42523_2020_48_MOESM1_ESM.zip › gut_microbiota_Supplemental data_r2.docx]

**Supplementary figures and tables**

**Seasonal Dynamics and Starvation Impact on the Gut Microbiome of Urochordate Ascidian *Halocynthia roretzi***

Jiankai Wei^1,2,3^, Hongwei Gao^4^, Yang Yang, Haiming Liu, Haiyan Yu, Zigui Chen^5,^*, Bo Dong^1,2,3,^*

^1^ Key Laboratory of Marine Genetics and Breeding, College of Marine Life Sciences, Ocean University of China, Qingdao 266003, China

^2^ Laboratory for Marine Biology and Biotechnology, Qingdao National Laboratory for Marine Science and Technology, Qingdao 266237, China

^3^ Institute of Evolution and Marine Biodiversity, Ocean University of China, Qingdao 266003, China

^4^ Technology Center of Qingdao Customs, Qingdao 266002, China

^5^ Department of Microbiology, Faculty of Medicine, The Chinese University of Hong Kong, Hong Kong, China

*Correspondence authors:

Bo Dong ([bodong@ouc.edu.cn](mailto:bodong@ouc.edu.cn))

Zigui Chen (zigui.chen@cuhk.edu.hk)

**
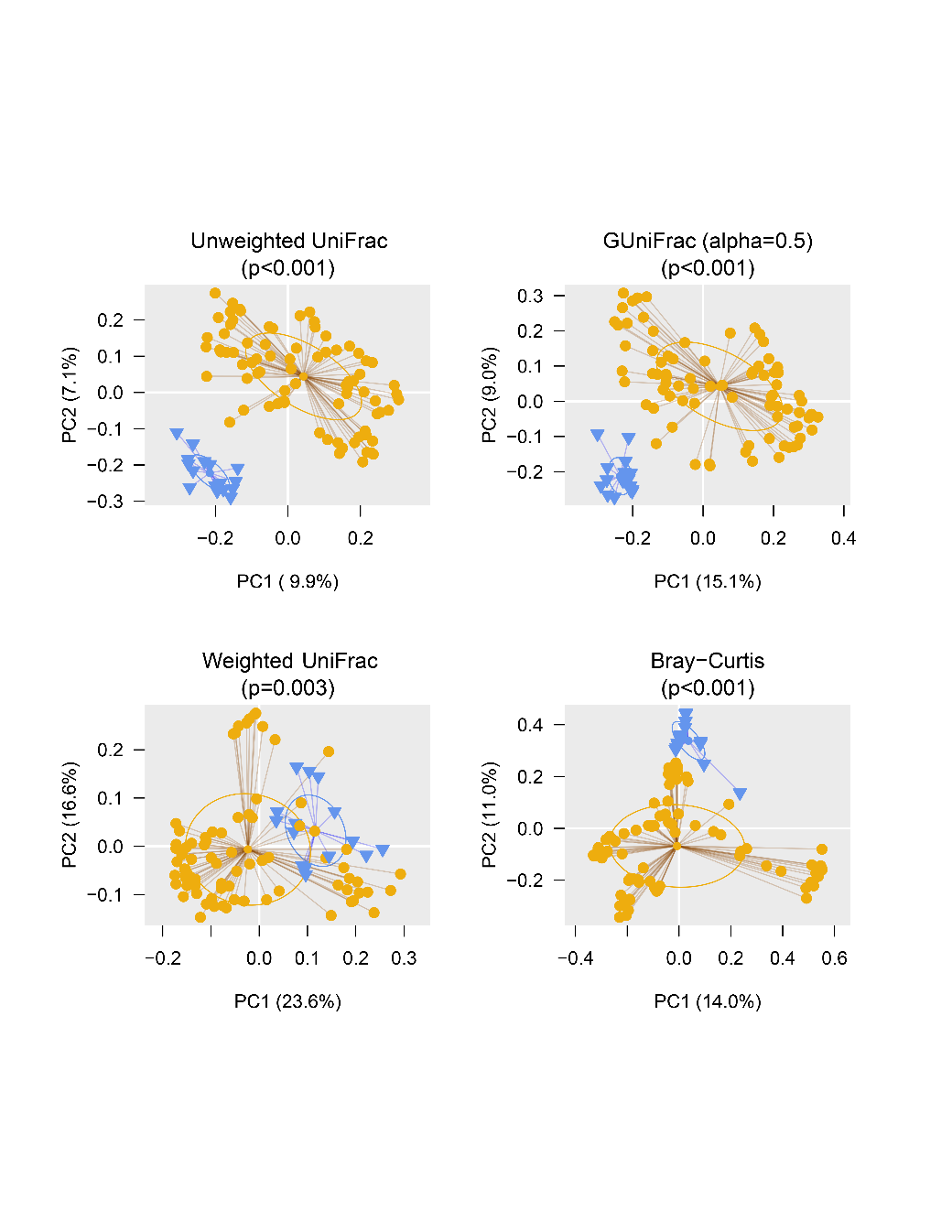
**

**Figure S1.** The clustering results of all the samples based on different principal component analysis of the 16S rRNA gene sequencing including Unweighted UniFrac, GUniFrac, Weighted Unifrac Bray-Curtis methods. The stool samples were labeled in yellow dots, while the sea water samples were labeled in blue triangles.


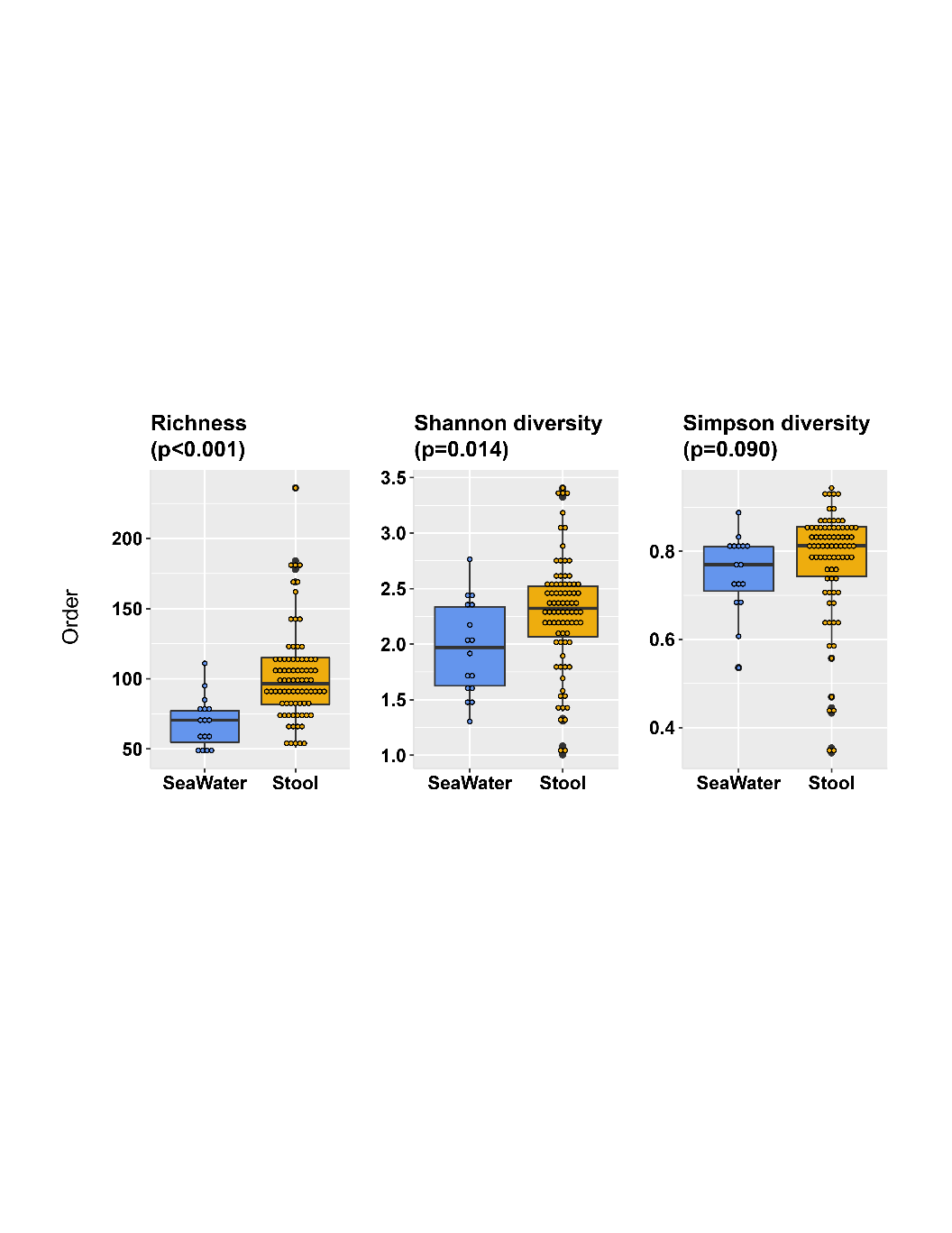


**Figure S2.** The box plot of the richness, Shannon diversity and Simpson diversity of seawater and stool samples at the order level. The stool samples showed significantly higher levels of richness and Shannon diversity.

**
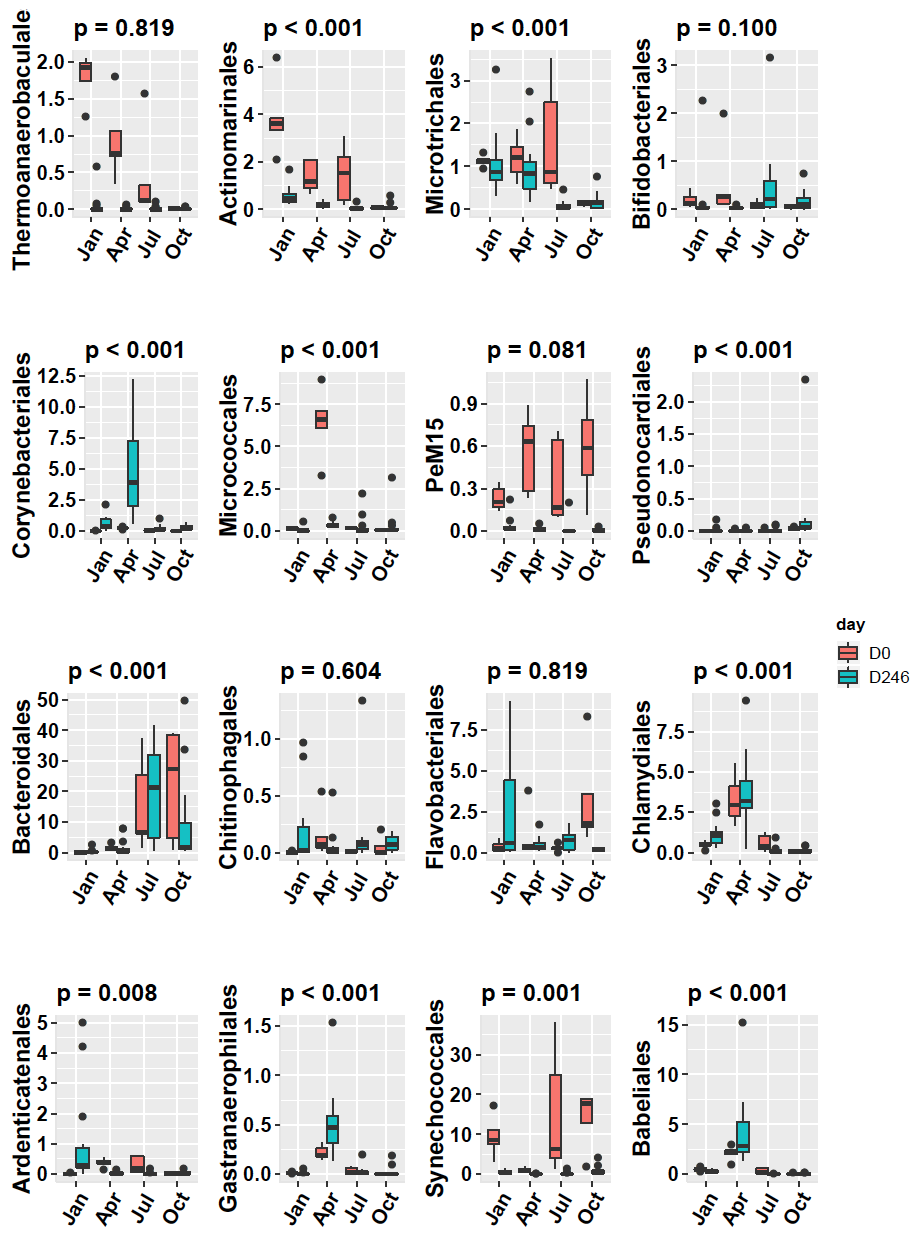
**

**
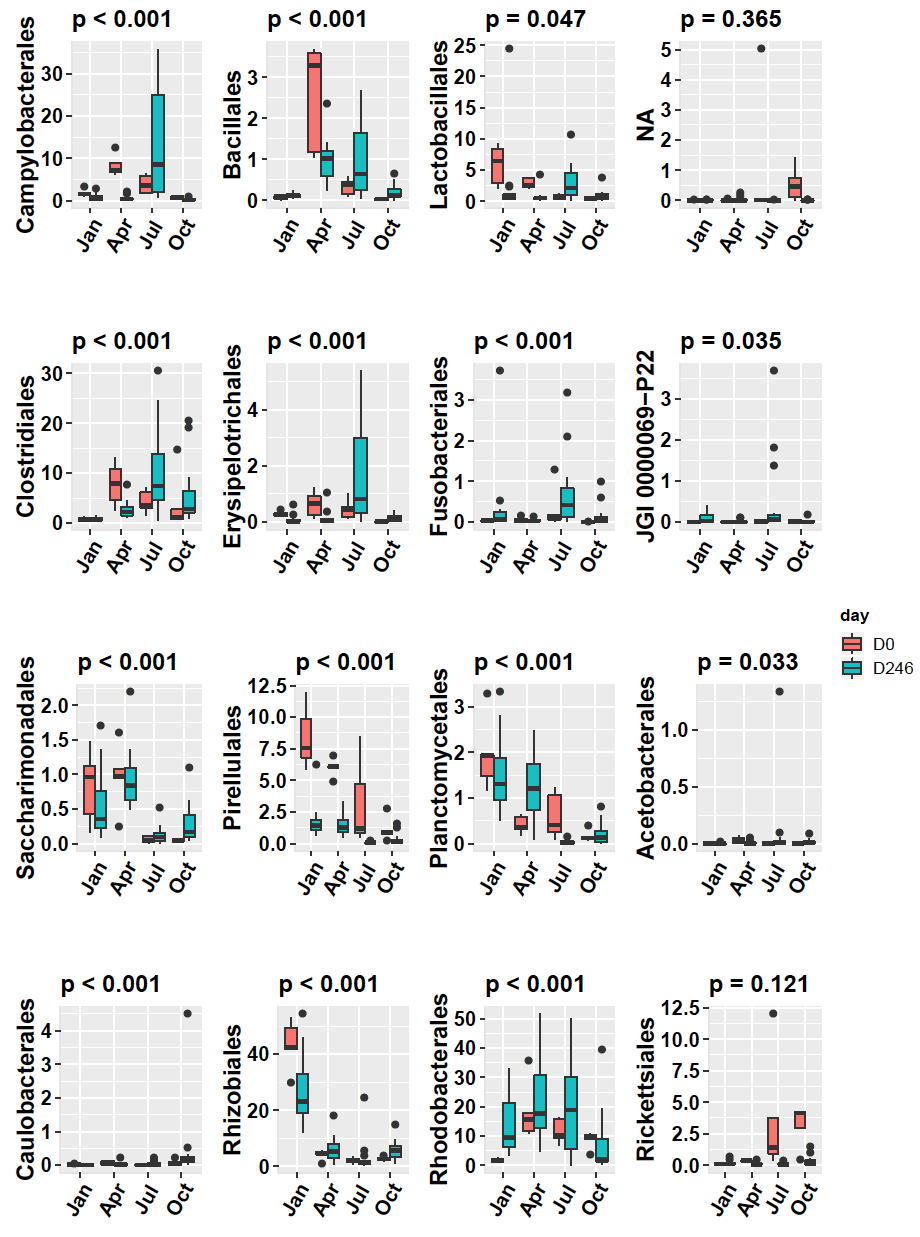
**

**
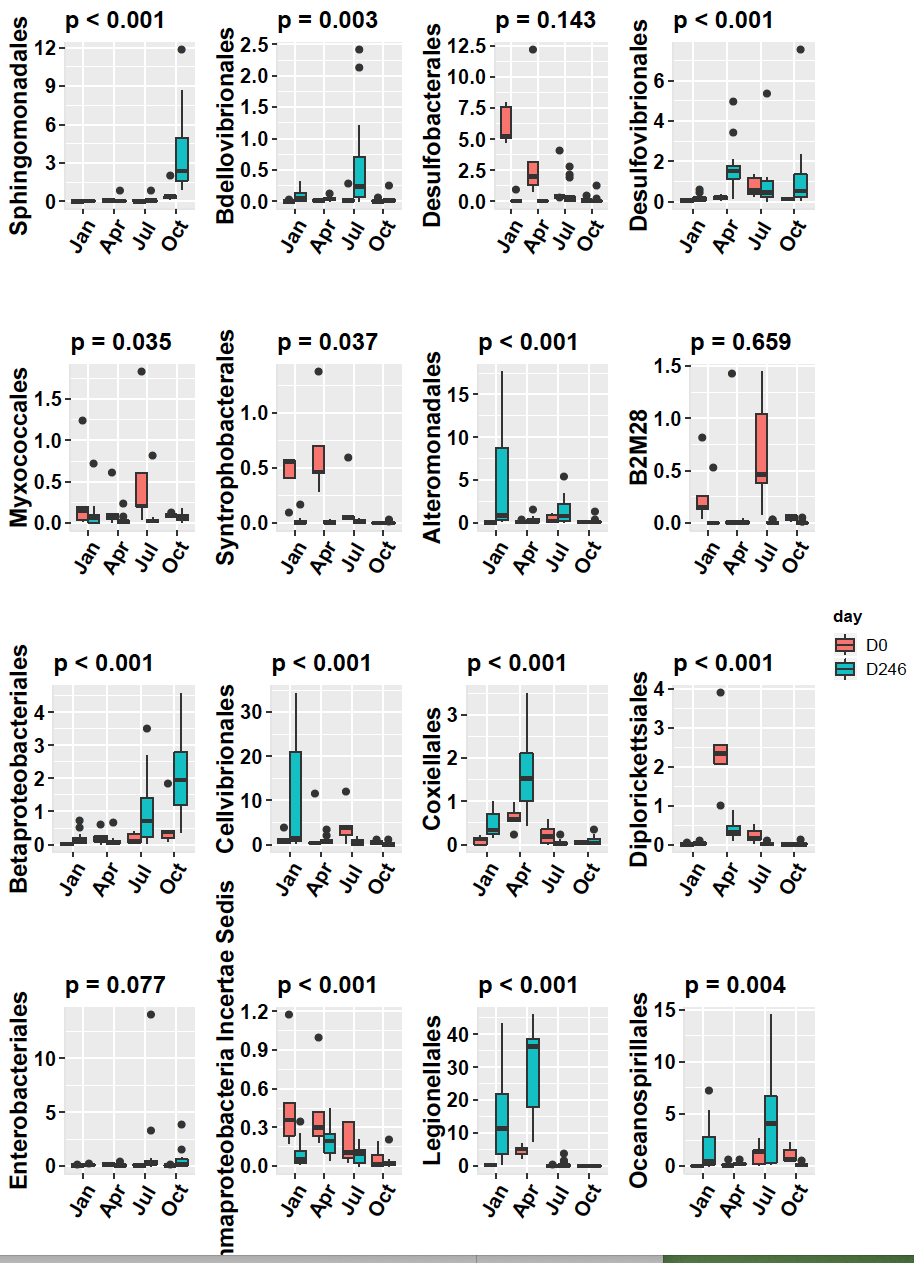
**

**
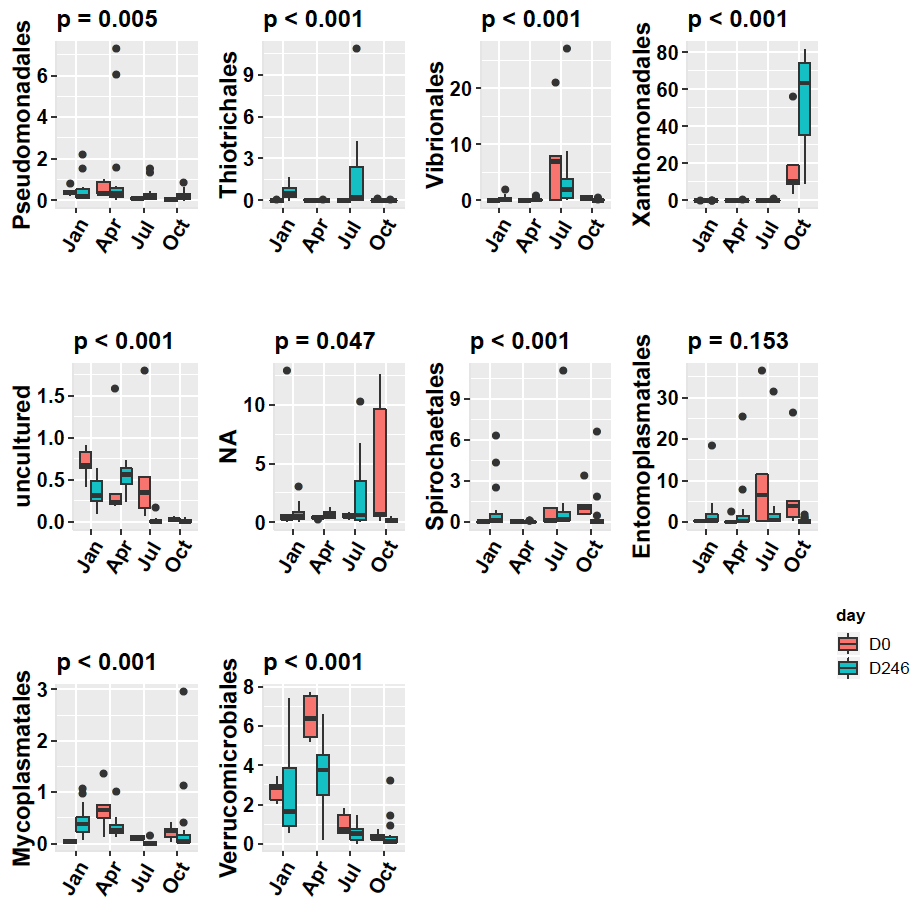
**

**Figure S3.** The bacteria abundance along with different seasons and starvation treatments at the order level. The samples were divided into four seasons according to the sampling date in the X axis. The Y axis indicated the relative abundance of each bacteria. The samples without starvation were labeled in red color and the samples with starvation were labeled in blue color in each column. The order names were labeled in the left of each column.

**
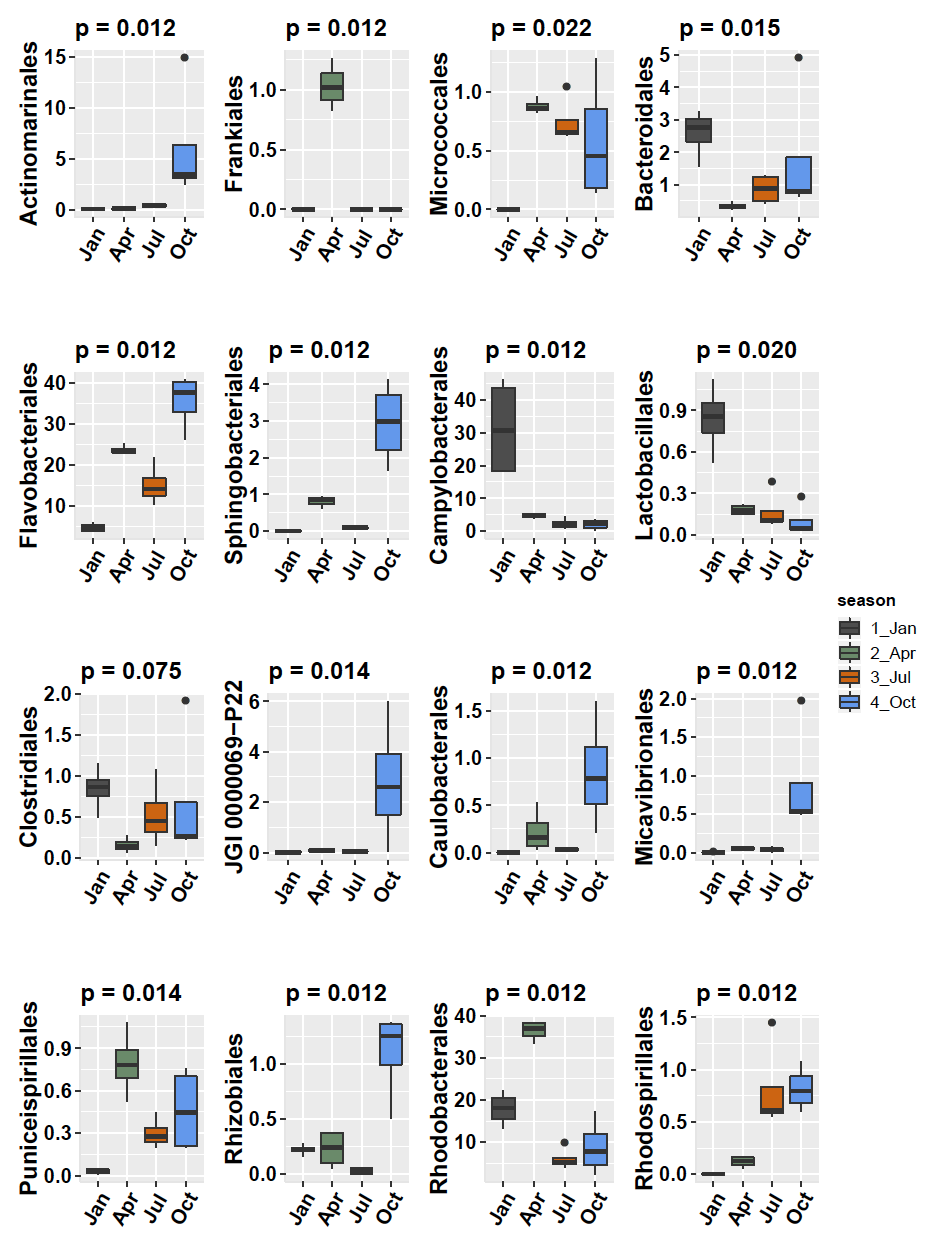
**

**
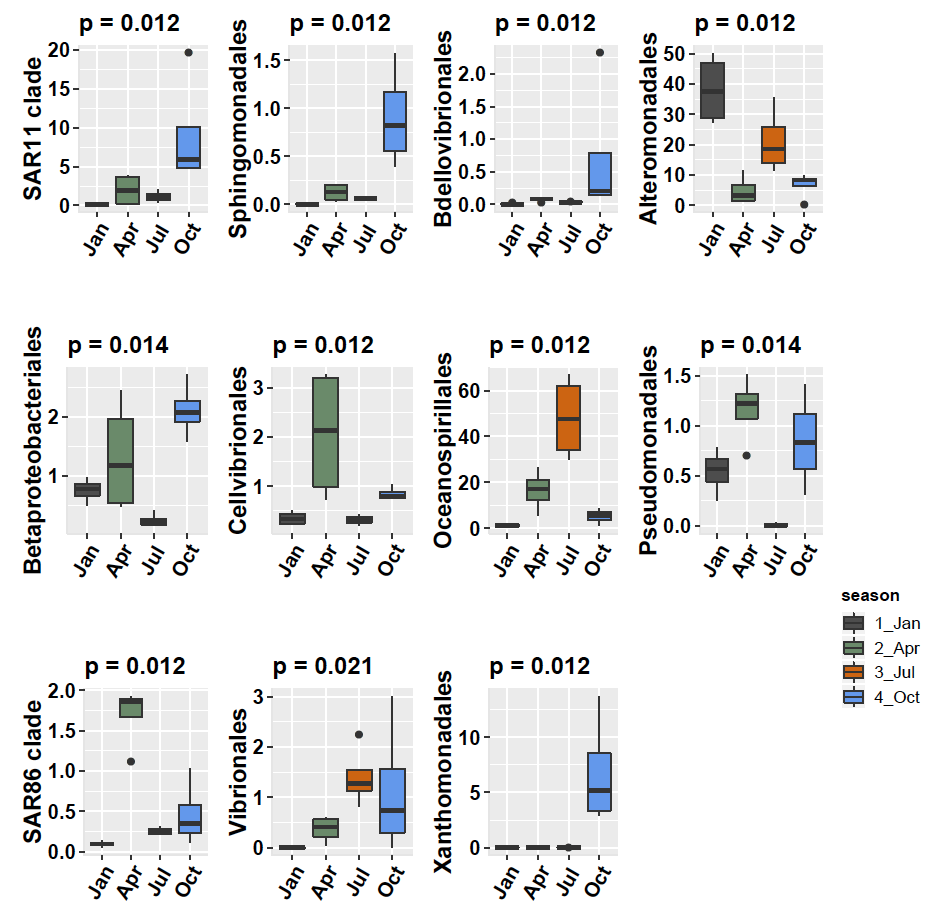
**

**Figure S4.** The bacteria abundance along with different seasons at the order level. The samples were divided into four seasons according to the sampling date and labeled in four different colors in X axis. The Y axis indicated the relative abundance of each bacteria. The order names were labeled in the left of each column.

**
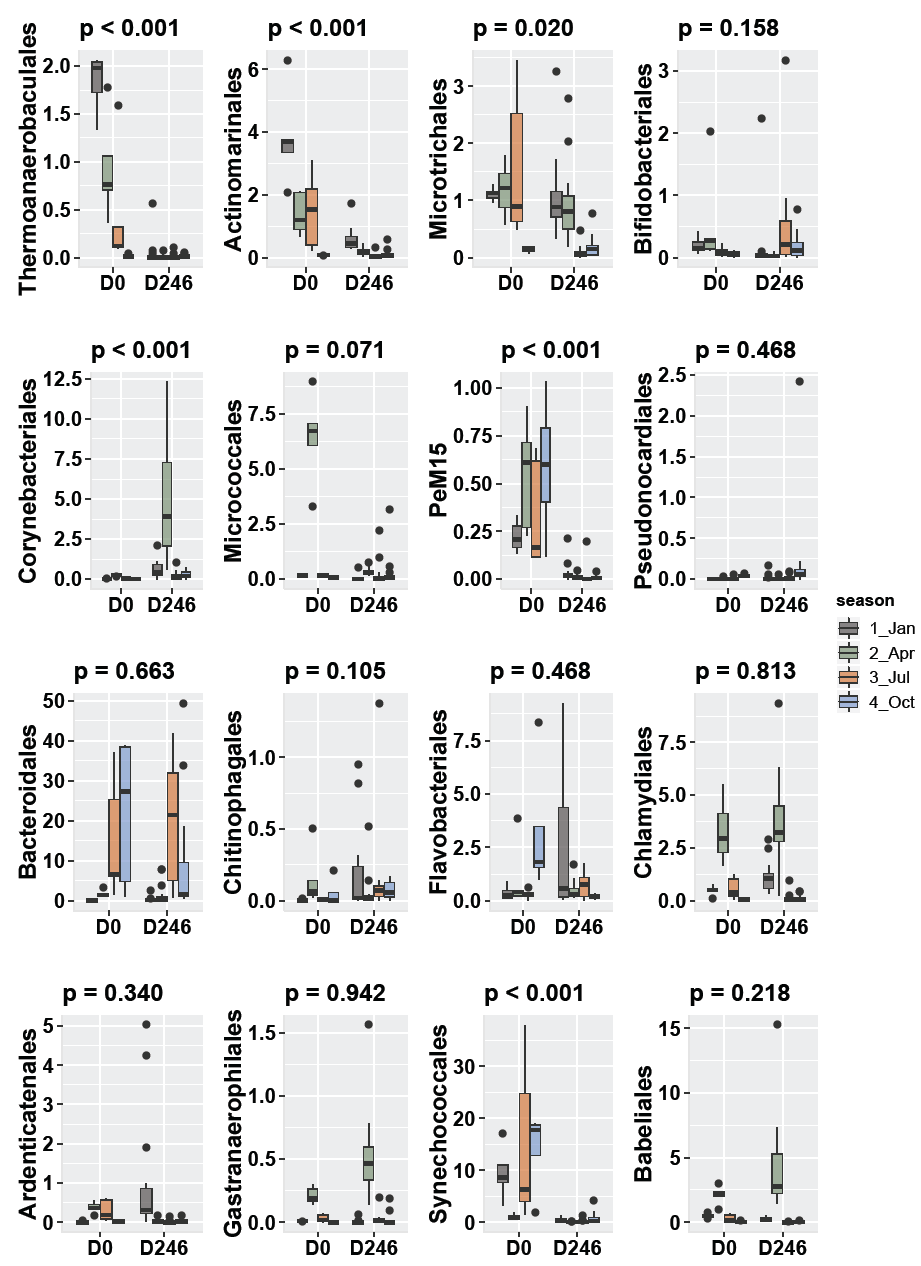
**

**
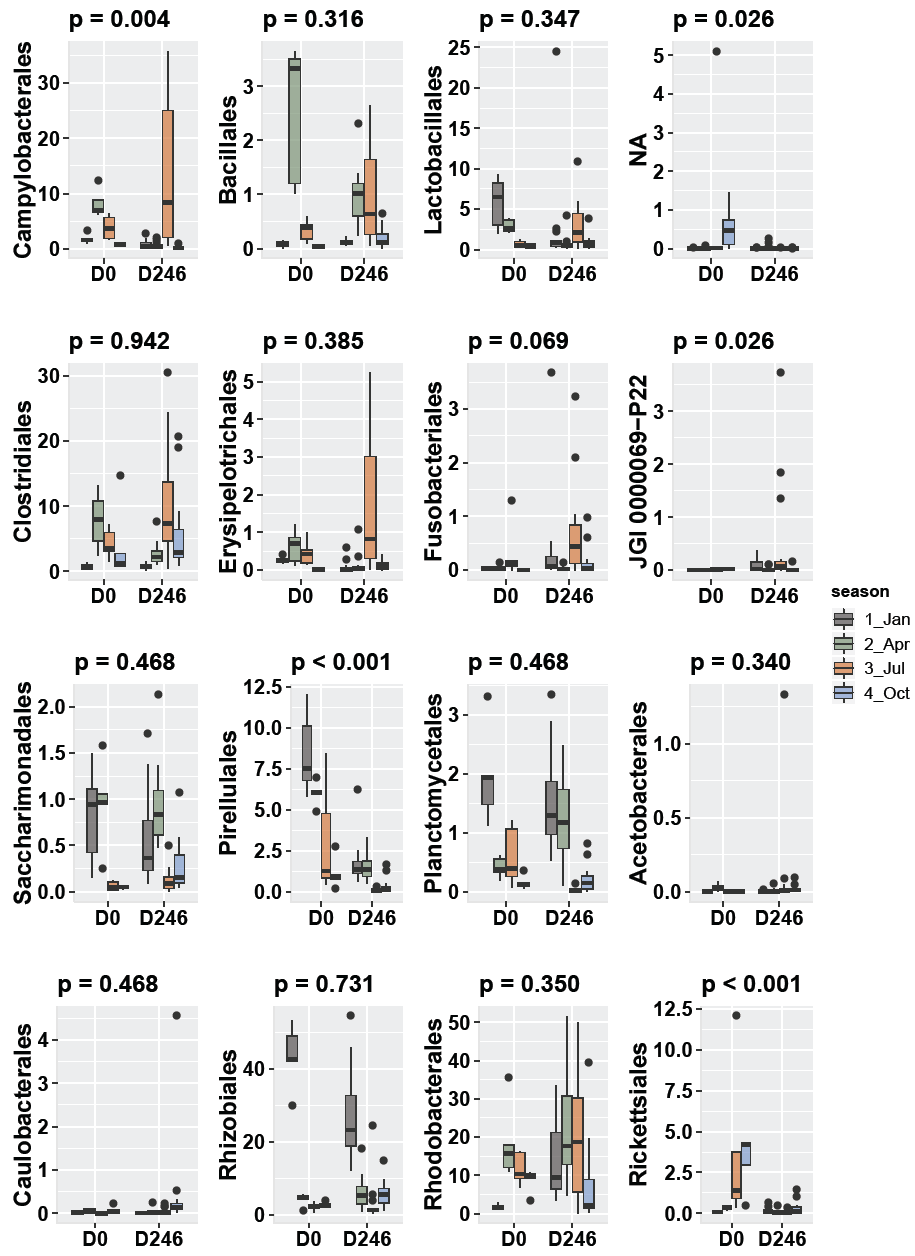
**

**
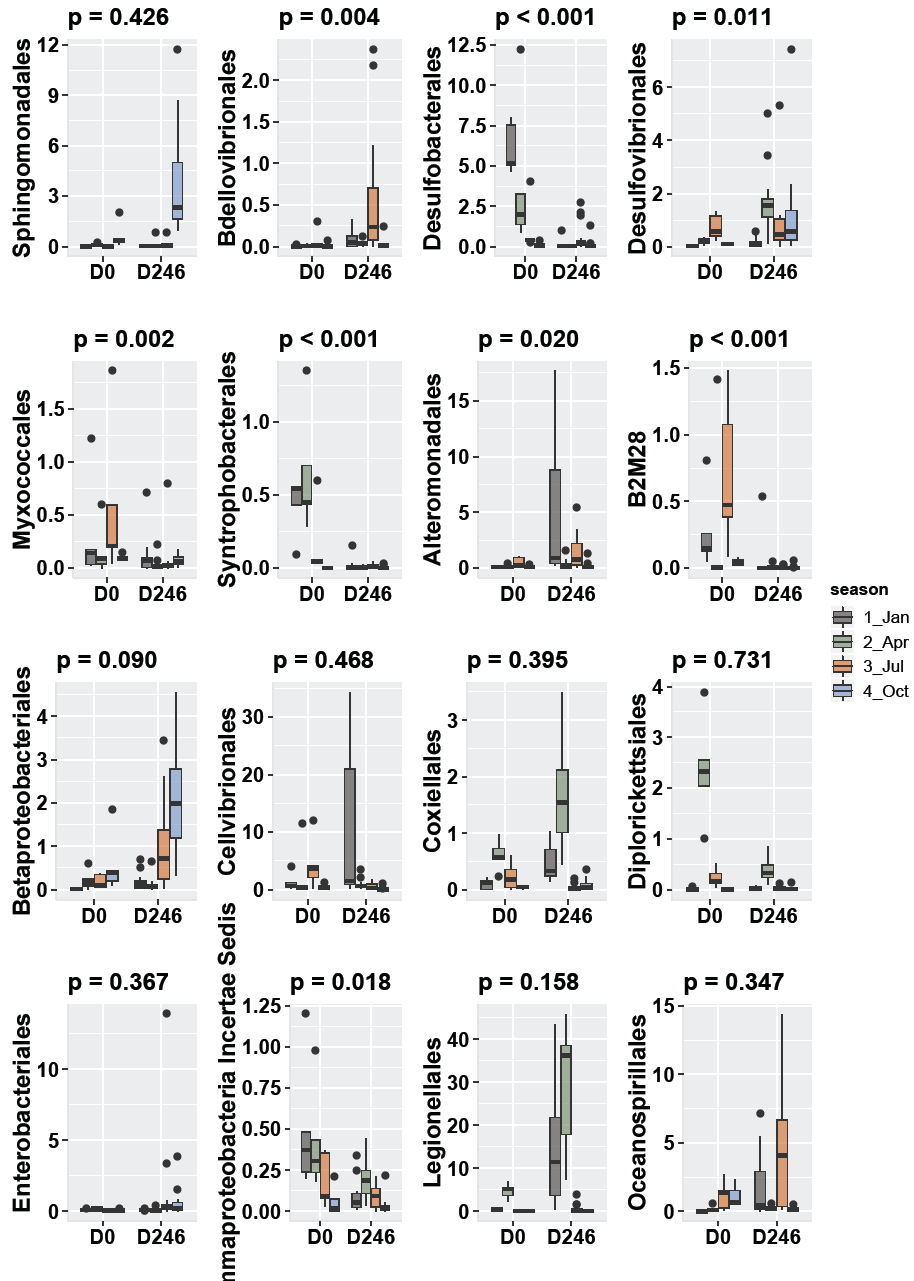
**

**
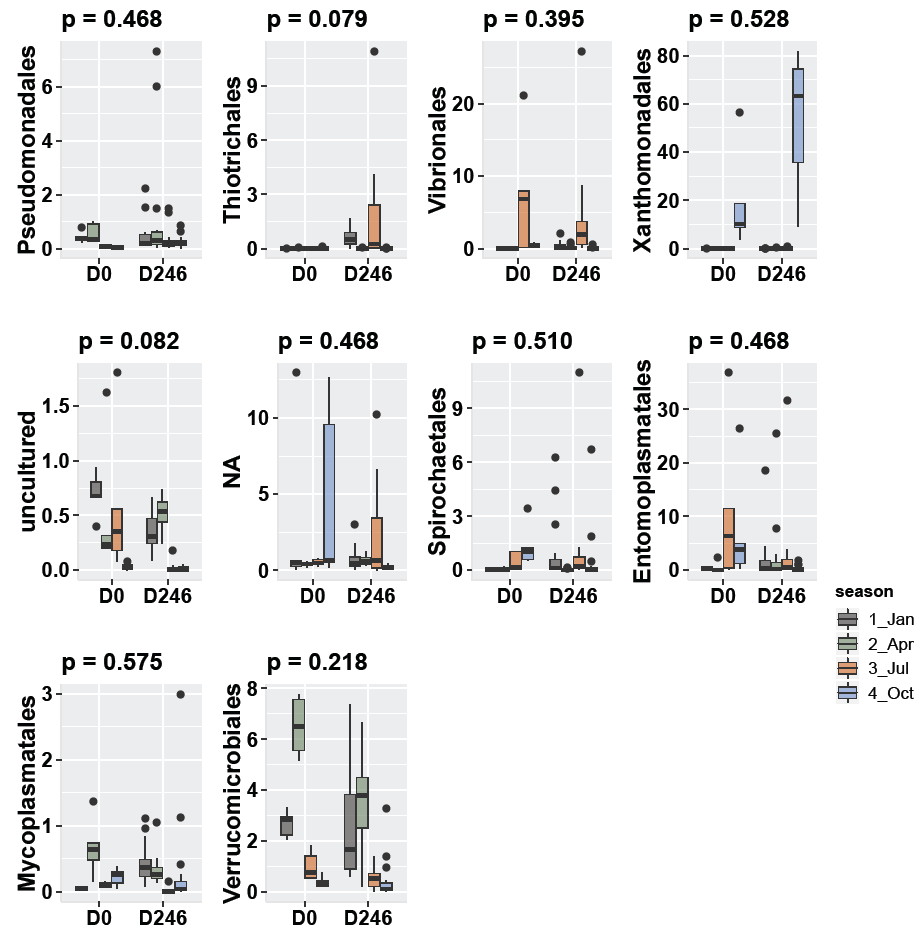
**

**Figure S5.** The bacteria abundance along with starvation treatments at the order level. The samples were divided into two stages according to the starvation treatments in X axis. The Y axis indicated the relative abundance of each bacteria. The samples in different seasons were labeled in four different colors in each column. The order names were labeled in the left of each column.


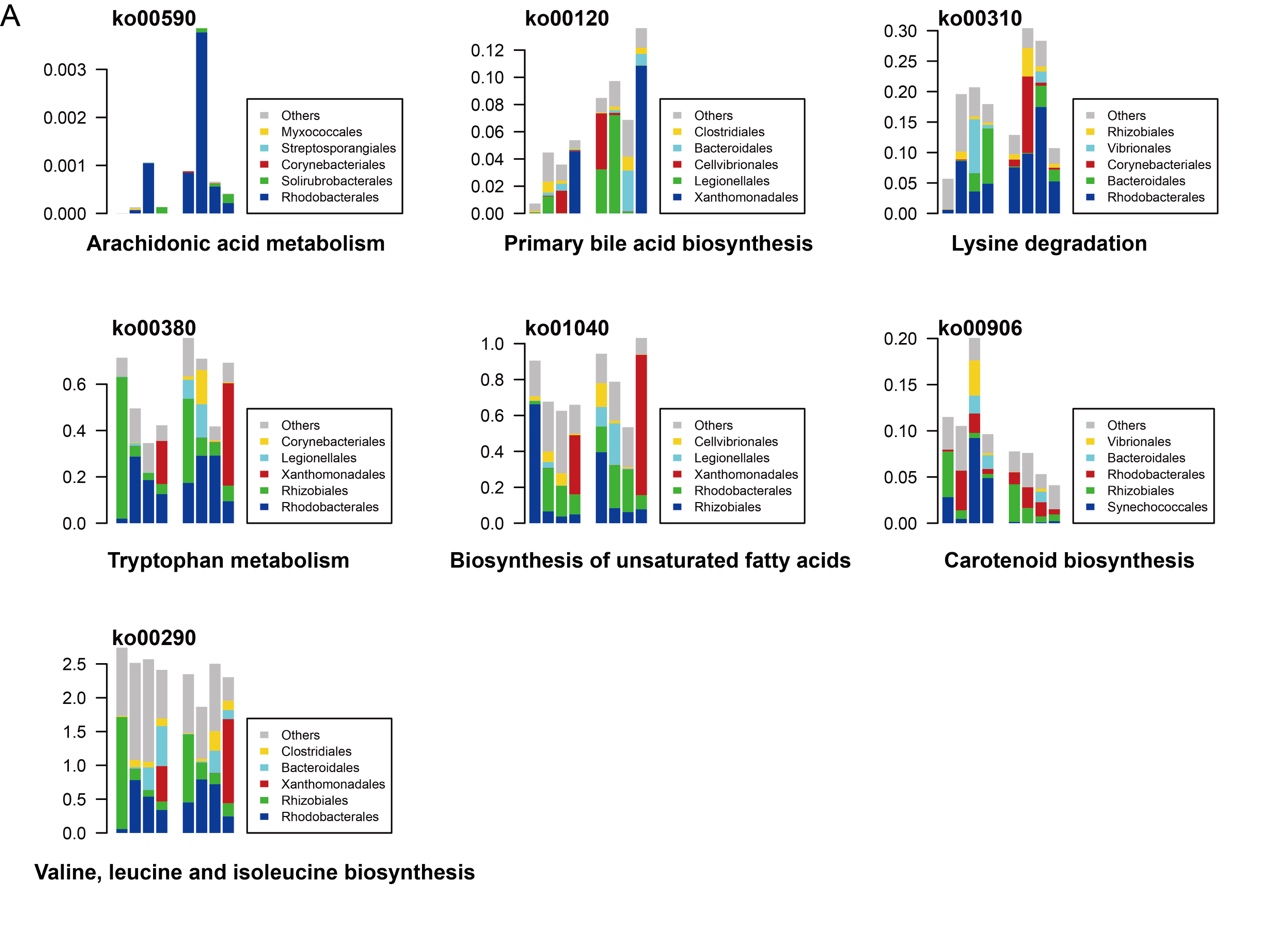


**Figure S6.** The discriminated KO pathways and the top 6 colonized bacteria before and after starvation. The x axis indicated different samples while the y axis indicated the relative abundance of the bacteria. The different colors indicated different bacteria orders and the order names were listed in the rectangular box. The ko pathway annotations were listed below the column.


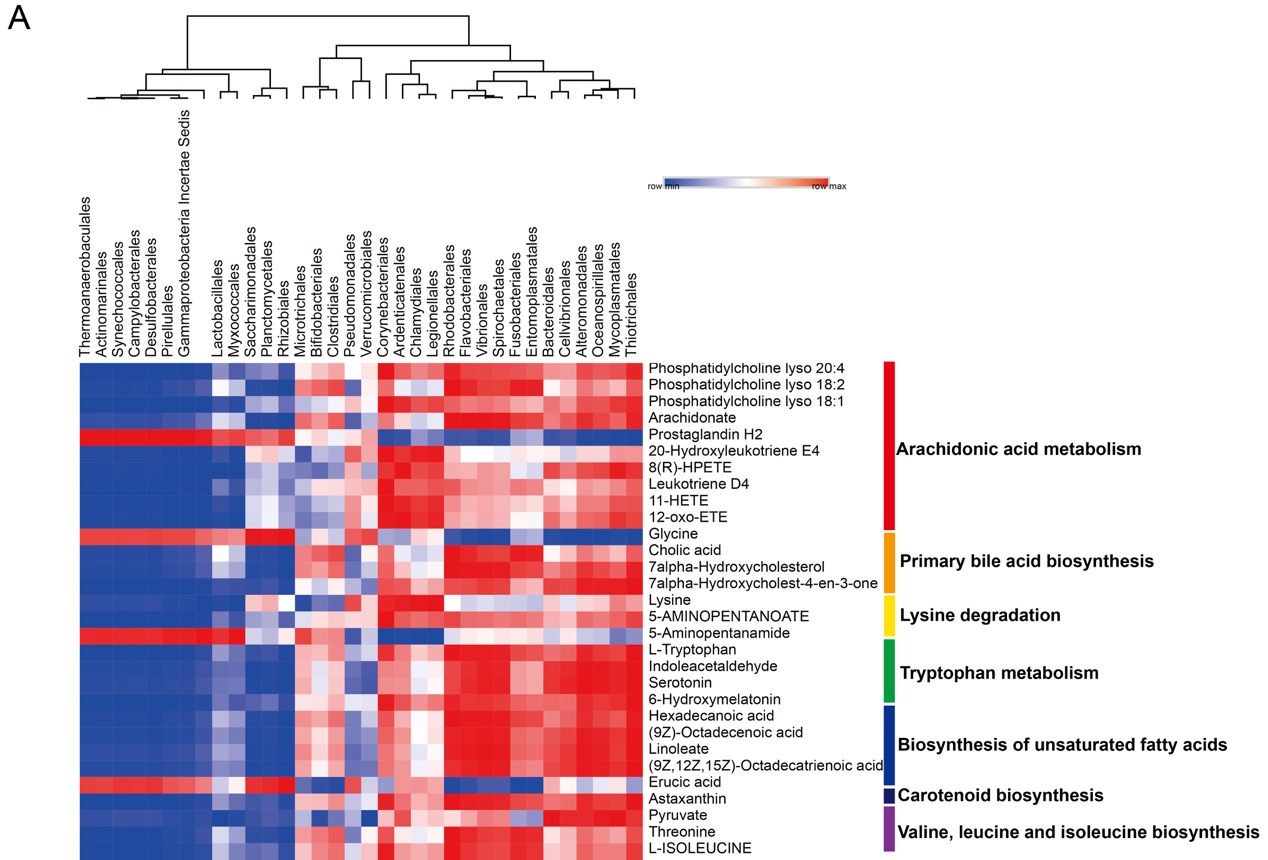


**Figure S7.** The Pearson correlation analysis of abundance between gut bacteria and metabolites from winter samples. The blue color indicated low correlation while the red color indicated high correlation. The order names of bacteria and the classification of metabolites were also listed.

**
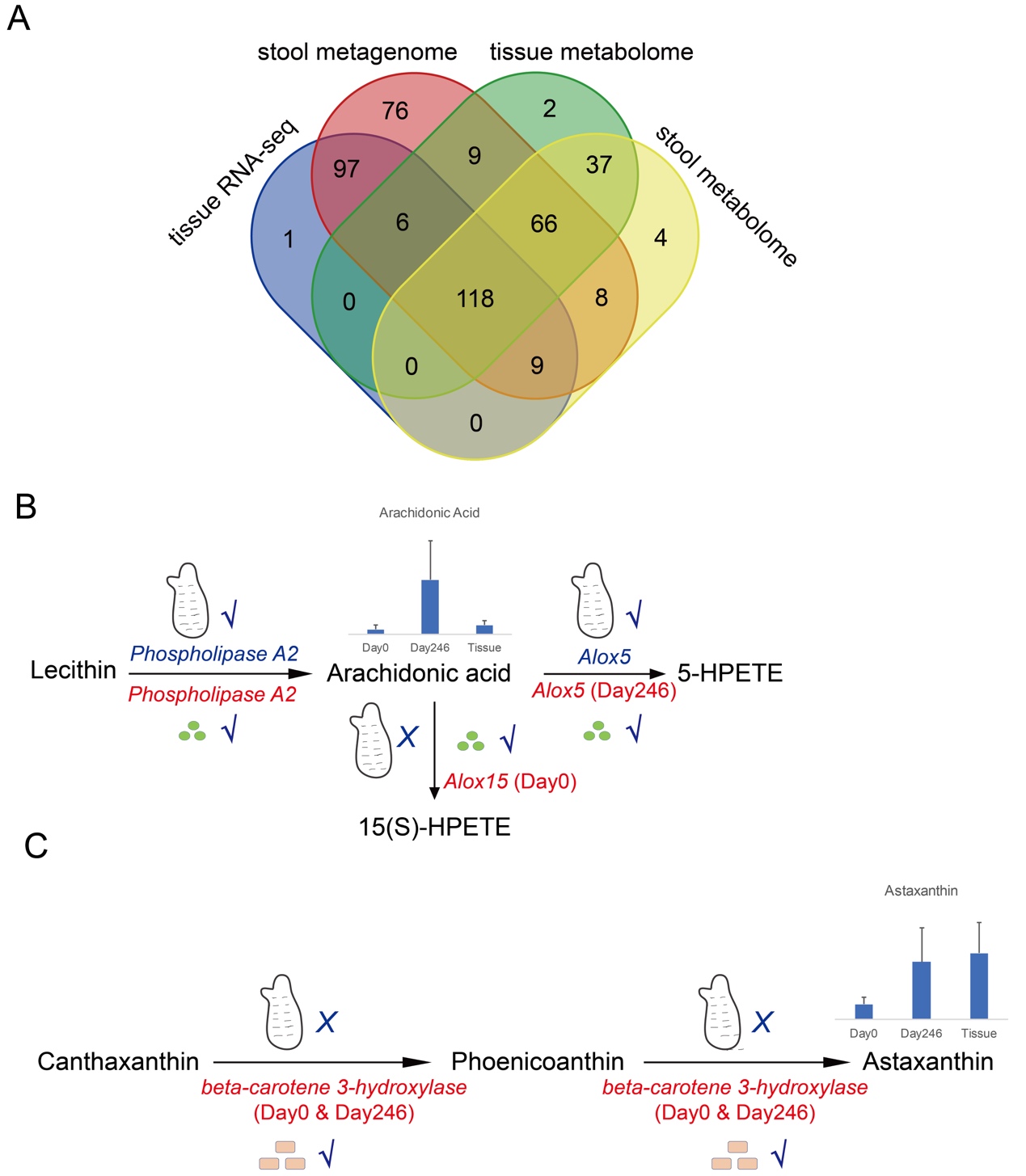
**

**Figure S8.** The metabolites source identification by gene annotations. (A) The KEGG pathway annotation overlaps among ascidian tissue RNA-seq data, stool metagenome data and metabolome data. (B) The metabolic process of arachidonic acid. The genes identified in ascidian transcriptomes were labeled in blue while the genes identified in bacteria metagenomes were labeled in red. The column indicated the abundance of arachidonic acid in different groups. (C) The synthesis process of astaxanthin. The genes were identified only in metagenomes. The column indicated the abundance of astaxanthin in different groups.

**Table S1.** Differential bacterial phylum between seawater and ascidian stool microbiota.

**Table S2.** Differential bacterial order between seawater and ascidian stool microbiota.

**Table S3.** Differential bacterial order along with seasons and starvation treatments in ascidian stool samples.

**Table S4.** Differential bacterial order along with seasons in seawater samples.

**Table S5.** Differential bacterial genus along with seasons and starvation treatments in ascidian stool samples.

**Table S6.** The discriminated KO pathways before and after starvation for each season.

**Table S7.** The metabolites identified from LC-MS experiment.

**Table S8.** The differentially expressed metabolites between different groups.

**Table S9.** The annotation of unigenes from transcriptomic sequencing against nr database and Swiss-Port database.

**Table S10.** The annotation of unigenes from metagenomic sequencing against Non-supervised Orthologous Groups (eggNOG) database.
